# Neurocognitive signatures of phonemic sequencing in expert backward speakers

**DOI:** 10.1038/s41598-020-67551-z

**Published:** 2020-06-30

**Authors:** María José Torres-Prioris, Diana López-Barroso, Estela Càmara, Sol Fittipaldi, Lucas Sedeño, Agustín Ibáñez, Marcelo L. Berthier, Adolfo M. García

**Affiliations:** 1 Cognitive Neurology and Aphasia Unit, Centro de Investigaciones Médico-Sanitarias, Instituto de Investigación Biomédica de Málaga (IBIMA), University of Malaga, Malaga, Spain; 2 Area of Psychobiology, Faculty of Psychology and Speech Therapy, University of Malaga, Malaga, Spain; 3 Cognition and Brain Plasticity Unit, Bellvitge Biomedical Research Institute (IDIBELL), L’Hospitalet de Llobregat, Barcelona, Spain; 4 Universidad de San Andrés, Vito Dumas 284, B1644BID Victoria, Buenos Aires, Argentina; 5 National Scientific and Technical Research Council (CONICET), Buenos Aires, Argentina; 6 Universidad Autónoma del Caribe, Barranquilla, Colombia; 7 Center for Social and Cognitive Neuroscience (CSCN), School of Psychology, Universidad Adolfo Ibáñez, Santiago, Chile; 8 Global Brain Health Institute, University of California, San Francisco, United States; 9 Faculty of Education, National University of Cuyo (UNCuyo), Mendoza, Argentina; 10 Departamento de Lingüística Y Literatura, Facultad de Humanidades, Universidad de Santiago de Chile, Santiago, Chile

**Keywords:** Language, Neural circuits

## Abstract

Despite its prolific growth, neurolinguistic research on phonemic sequencing has largely neglected the study of individuals with highly developed skills in this domain. To bridge this gap, we report multidimensional signatures of two experts in backward speech, that is, the capacity to produce utterances by reversing the order of phonemes while retaining their identity. Our approach included behavioral assessments of backward and forward speech alongside neuroimaging measures of voxel-based morphometry, diffusion tensor imaging, and resting-state functional connectivity. Relative to controls, both backward speakers exhibited behavioral advantages for reversing words and sentences of varying complexity, irrespective of working memory skills. These patterns were accompanied by increased grey matter volume, higher mean diffusivity, and enhanced functional connectivity along dorsal and ventral stream regions mediating phonological and other linguistic operations, with complementary support of areas subserving associative-visual and domain-general processes. Still, the specific loci of these neural patterns differed between both subjects, suggesting individual variability in the correlates of expert backward speech. Taken together, our results offer new vistas on the domain of phonemic sequencing, while illuminating neuroplastic patterns underlying extraordinary language abilities.

## Introduction

When visiting the local barber shop, a native of La Laguna, Spain, may be surprised by the speech of some fellow citizens. He might be greeted with the utterance *nasbue chesno* and fail to understand it at all. With time, however, he may realize that the phrase was a backward rendition of *buenas noches* (*good evening*) and that this peculiar way of speaking is quite widespread in this town. So much so, in fact, that a group of citizens demand that UNESCO acknowledge their linguistic extravaganza as intangible cultural heritage. Still, theirs might be a lost cause. Although word inversion is also part of other sociolects, such as Argentine *lunfardo*, the Canary Academy of Language has declared that this phenomenon has no scholarly value. Yet, that position is arguably short-sighted. Backward speech constitutes an extraordinary ability to quickly reverse words, pseudowords, and even sentences, which requires reordering phonemes while retaining their identity. Therefore, it offers a useful model to study phoneme sequencing as a key stage of phonological-phonetic encoding—i.e., the capacity to select, retrieve, and temporarily arrange phonemic information to set up an articulatory plan^1,2^. Based on this premise, the present study offers a novel approach to phonemic sequencing mechanisms by examining behavioral, neuroanatomical, and neurofunctional signatures of expertise in backward speech.

Pioneering behavioral research on phoneme sequencing was mainly concerned with speech errors in healthy^3–5^ and brain-damaged^6–8^ subjects, giving rise to serial associative, frame-based, and dynamic control signal psycholinguistic models^3^. Although neuroimaging studies prove less widespread, phoneme sequencing mechanisms have also been described in neurocognitive models^9^, with most evidence pointing to an extended network including the inferior frontal gyrus (IFG)^10–13^, the anterior insular cortex (IC)^12^, the supplementary motor area^12^, and the supramarginal gyrus (SMG)^11^. More generally, these findings are compatible with the dual-stream model of language^14,15^. This leading account posits that phonological encoding would be mainly subserved by the dorsal stream, encompassing temporo-frontal areas directly connected through the long segment of the arcuate fasciculus (AF) and by two indirect segments: a parieto-temporal segment (AF posterior segment) and a parieto-frontal one (AF anterior segment)^16^. In particular, the dorsal stream would be crucial for phoneme sequencing control, affording internal speech feedback via predictions and updates about the state of articulators and the sensory consequences of the motor program deployed^9^. Indeed, damage to dorsal cortical regions^17^, including the superior temporal gyrus (STG) and the SMG^18^, the precentral and postcentral cortices^19^, and the underlying white matter^18,20^, correlates with the frequency of phonemic paraphasias (misplacement of phonemes) and neologisms in aphasic patients. Conversely, other linguistic functions, including lexico-semantic access, would be critically supported by the ventral stream^14,15^, comprised of temporo-frontal areas connected through the inferior longitudinal fasciculus (ILF), the inferior fronto-occipital fasciculus (IFOF), and the uncinate fasciculus (UF)^21^. This stream would facilitate processing familiar phonemic sequences as chunks through a direct lexical-motor system, requiring less segment-by-segment supervision^9^. Moreover, the ventral stream would offer secondary contributions to core phonological processes, especially when dorsal regions are overloaded^22^ or otherwise compromised^23–26^.

Yet, multidimensional neurocognitive studies on phonemic sequencing are scant^12^. In particular, no such study has examined the issue targeting expert backward speakers, who represent strategic models of the domain given their elevated phoneme sequencing skills. This paves the way for novel research in the field.

Backward speech can manifest in different forms depending on the reversed unit. At word level, reversal may be done by rearranging phonemes (e.g., *basket* /bæskɪt/ becomes teksab/tɪksæb/) or syllables (e.g., *basket* /bæskɪt/ becomes *ketbas*/kɪtbæs/)^27^. At sentence level, these operations may be performed while constituents are also reversed (i.e., starting from the last word and going backwards to the first one, such that *basket is fun* /bæskɪt ɪs fʌn/ becomes *nuf si teksab*/nʌf sɪ tɪksæb/) or while their syntactic ordering is preserved within the phrase (e.g., *basket is fun* /bæskɪt ɪs fʌn/ becomes *teksab si nuf* /tɪksæb sɪ nʌf/)^27^. Both forms of backward speech may be supported by enhanced inner visualization of written words or phrases^27–30^ or heightened working memory abilities^30,31^. Accordingly, elevated word reversal skills could be mediated by the capacity to store and (re)order verbal or visual information^31^. Of note, backward speech proves particularly feasible in languages with transparent or relatively transparent orthography, such as Spanish^32^, as this allows for phonemes to nearly always retain their identity (same sound) irrespective of their position and surrounding segments.

Although a number of studies have examined linguistic aspects of backward speech in healthy subjects^27,28,30,33–35^, evidence on its neural signatures proves markedly scant. The few case studies on pathological backward speech^29,36,37^ provide vague references to frontal or temporal lesions, without further neural specification. More relevantly, the only study exploring in vivo neural correlates of this skill (in a healthy backward speaker with exceptional working memory abilities) showed greater activity during backward than during forward speech mainly in the bilateral IFG and STG, as well as the left SMG, superior parietal cortex, and fusiform gyrus (FFG)^31^. Of note, these areas are implicated in phonological processing and verbal working memory^31,38^, with the FFG playing an additional role in visual imagery^39^. This evidence suggests that backward speech (and with it, phonemic sequencing skills) would critically rely on dorsal stream regions, with less critical involvement of the ventral stream^14,15^. However, no study has systematically examined this issue, let alone integrating neuroanatomical and functional connectivity methods.

Aiming to characterize core mechanisms of expert phonemic sequencing, we examined behavioral, neuroanatomical, and neurofunctional signatures of backward speech in two highly skilled subjects relative to matched controls. First, we tested the participants’ outcomes in backward and forward speech tasks. Also, in light of previous works, we assessed their relationship with working memory abilities via simple and complex span tasks, and explored whether reversed words are processed as lexical or non-lexical entries via a lexical decision task^40^. Second, to identify whether the backward speakers presented a distinctive neural configuration, we conducted multimodal neuroimaging analyses, encompassing voxel-based morphometry (VBM), diffusion tensor imaging (DTI), and fMRI-derived resting-state functional connectivity (rsFC). Specifically, we addressed the following questions: (1) which are the key brain signatures of backward speech expertise?; (2) are they evenly traceable across the dorsal and ventral streams?; and (3) are these signatures consistent across backward speakers? Considering previous evidence on phoneme sequencing mechanisms and insights from other expert language users (simultaneous interpreters and phoneticians), backward speakers can be expected to show (a) distinctive gray matter volume (assessed via VBM) in areas subserving sound-to-articulation mapping and motor speech control (e.g. IFG, IC, pSTG); (b) microstructural white matter differences (indexed by fractional anisotropy [FA] and/or mean diffusivity [MD] metrics) in the AF; and (c) enhanced rsFC patterns between dorsal areas and other regions subserving linguistic or domain-general control functions. However, based on previous evidence^22–26^ and considering the high phonemic demands of sustained backward speech, complementary contributions of ventral stream areas and tracts (ILF, IFOF, and UF) could be expected in both subjects. Briefly, building on this multidimensional approach, the present study seeks to offer novel insights on the neural underpinnings of phonological sequencing skills.

## Methods

### Participants

The study focused on two native Spanish speakers with an exceptional ability to reverse words and sentences, together with a sociodemographically matched control group. Backward speaker 1 (BS1) was a 43-year-old left-handed man with 17 years of formal education who worked as a system engineer. He had normal hearing and vision. Except for a period of developmental stuttering between ages 10 and 12, he reported no history of other learning difficulties, psychiatric conditions or neurological disorders. BS1 realized that he could easily reverse words at age 14, in the absence of any explicit learning on this skill. Throughout his adulthood, he has constantly made deliberate (non-pathological) use of backward speech daily without resorting to any conscious strategy. For reversing words, he would rearrange each phoneme from last to first. For instance, he reversed the word *banana* /banana/ as *ananab*/ananab/. He stated that he typically reverses simple words effortlessly, whereas long words (e.g., *structuralism*) or long sentences prove more demanding. Notably, when faced with a sentence, he states that he can opt to reverse all words while keeping or reversing their ordering. Yet, for the purpose of assessment, he was asked to reverse from the last to the first word. None of BS1’s relatives have ever presented this ability.

Backward speaker 2 (BS2) was a 50-year-old right-handed man with 14 years of formal education who worked as a photographer. His hearing and vision were normal, and he reported no history of learning difficulties or neuropsychiatric disorders. He effortlessly developed the ability to voluntarily reverse speech at age 8. Although he uses backward speech frequently, he has never explicitly practiced this skill. As in the case of BS1, his reversals operate on the phonemic (rather than the graphemic) level. His backward speech was also based on the words’ phonological structure but, when reversing sentences, he only reversed them from the last to the first word. He reported that he cannot identify any specific, conscious strategy supporting his backward speech, although he declared being able to internally “see” flashes of the written words. The only family member with a similar skill was his 19-year-old daughter, although our behavioral assessment revealed that her performance was substantially poorer than BS2’s (unpublished data).

The control sample for the behavioral study comprised 18 healthy men with a mean of 38.9 (*SD* = 10.9) years of age and an average of 15.7 (*SD* = 1.8) years of education. All subjects showed normal hearing and normal or corrected-to-normal vision, and reported no history of learning difficulties, psychiatric or neurological disorders, or drug or alcohol abuse. This sample was matched with both experimental subjects in terms of age (BS1: Crawford’s *t*, two-tailed = 0.277; *p* = 0.785; BS2: Crawford’s *t*, two-tailed = 0.902; *p* = 0.379) and education level (BS1: Crawford’s *t*, two-tailed = 0.703; *p* = 0.491; BS2: Crawford’s *t*, two-tailed = − 0.919; *p* = 0.370).

A different control group composed by 24 participants was used for the neuroimaging analyses, yet the number of participants included in each analysis varied (see “Neuroimaging data acquisition” section). All control participants were healthy men meeting the same inclusion and exclusion criteria detailed above. These participants had a mean age of 32 years old (*SD* = 15.1) and an average of 15 (*SD* = 3.1) years of education. The whole sample was matched with both subjects for age (BS1: Crawford’s *t*, two-tailed = 0.715; *p* = 0.482; BS2: Crawford’s *t*, two-tailed = 1.169; *p* = 0.254) and education level (BS1: Crawford’s *t*, two-tailed = 0.632; *p* = 0.533; BS2: Crawford’s *t*, two-tailed =  − 0.316; *p* = 0.754).

The study was approved by the Ethics Committee of the Institute of Cognitive Neurology (Buenos Aires, Argentina). All participants provided written informed consent in accordance with the Declaration of Helsinki.

### Behavioral assessment

The behavioral assessments comprised two sets of tasks, targeting general cognitive skills as well as backward and forward language abilities, all detailed below. Testing was conducted in a quiet room over a single session lasting roughly 2 h per subject. All evaluations were performed by the same examiner. The outcomes of each backward speaker were compared to those of controls via Crawford’s two-tailed *t*-tests^41^. To control for the impact of working memory capacity, we used the BTD_Cov program^42^, which allows comparing the performance of a single subject with a multi-participant sample, controlling for the effect of covariates (in this case, the outcomes of the working memory tasks). Also, between-subjects and within-subject comparisons of accuracy were performed via Chi-squared tests (with Yate’s correction). Within-subject comparisons of response times (RTs) to different stimulus types in the lexical decision task were performed via two-tailed *t*-tests on SPSS software, version 25 (IBM, Armonk, NY, USA). In all analyses, alpha levels were set at *p* < 0.05.

#### General cognitive tasks

##### Reasoning test

To rule out the potential impact of general cognitive ability, we tested the participants’ non-verbal reasoning skills through the Matrix Reasoning subtest from the Wechsler Adult Intelligence Scale-III (WAIS-III)^43^. In each trial, participants were presented through a PC screen with an incomplete array of shapes and had to point to one out of six possible pieces to complete the pattern. The test consists of 26 items, scoring 1 point for each correct answer and 0 for incorrect ones. Raw scores were converted into standard scores with a maximum possible score of 19.

##### Memory tasks

Given that backward speech performance has been related to working memory abilities^30,31^, we compared verbal and visuospatial working memory skills between the backward speakers and controls, and assessed their potential impact on reversal abilities. To this end, we used two validated digit span tasks (backward and forward) and two complex span tasks (i.e., operational and symmetry span tasks)^44^. The use of two complex span tasks is strategic. Given that the operational span task hinges on verbal information while the symmetry span task involves spatial information^45^, their joint use allows testing the potential influence of working memory skills on backward speech outcomes while covering two relevant processing and storage modalities.

Forward and backward digit span: These tasks were taken from WAIS-III^43^. In the forward digit span task, subjects were required to repeat a sequence of numbers in the same order as the examiner. Stimuli were read aloud by the examiner. The number of digits presented ranged from two to nine, with two trials for each array, yielding a total of 16 trials. The maximum possible score was 16, with one point for each correct trial. For its part, the backward digit span task required subjects to repeat the sequence of digits in reverse order. The number of digits presented ranged from two to eight, with two trials for each number of digits (i.e., two sequences of two digits, two sequences of three digits, and so on), with a total of 14 trials. The maximum possible score was 14, with one point for each correct sequence. The task was interrupted if the subject could not complete any of the two trials with the same number of items. Given that typified age-adjusted scores are only available for the digit summed score (i.e., forward plus backward), raw scores were used.

Operational span task: This task measures working memory storage-plus-processing capacity^44,46^ using letters as to-be-remembered stimuli and math operations as the distractor task. Scores were calculated by summing the number of letters recorded correctly and in the right order (number of correct responses) and summing up the number of mistakes in math operations (speed and accuracy), named *math errors*^44^. As in previous reports, only subjects with a performance of 85% or higher in the math operations entered the analysis^44^ (for more details, see Supplementary Information, Sect. 1). This task was presented through E-prime 2.0 running on a Windows 7 PC.

Symmetry span task: The symmetry span task was used to measure working memory storage-plus-processing capacity^44,46^ when employing visuo-spatial information. This test resembles the operational span task but, in this case, participants were required to remember locations within a grid and judge whether a figure was symmetrical in its vertical axis or not as distractor. Two scores were calculated, namely: the number of correct positions and of symmetry errors. The former was obtained by summing up the number of locations remembered correctly and in the right presentation order; the latter was established by adding the number of accuracy and speed errors committed in the symmetry judgment trials. The criterion to determine speed errors was the same as that used for the operation span task but based on the time employed in the symmetry judgment trials (for more details, see Supplementary Information*,* Sect. 1). This task was run on the same software and hardware as the previous one.

#### Backward and forward language tasks

Word reversal abilities were examined through backward repetition tasks using stimuli of different types and length. Forward repetition conditions were used as control in each case. Moreover, to explore the role of different processing routes in backward speech outcomes, we administered a lexical decision task including words written in the prototypical form (i.e., typical lexical entries), words written in reversed direction (i.e., real words presented with a non-lexicalized phonemic arrangement), and pseudowords (i.e., non-lexical items). This task aimed to explore whether reversed words were treated as lexical entries or as non-lexical entries, depending on whether their outcomes were closer to those of real words or pseudowords.

Forward and backward word and pseudoword repetition: In these tasks participants were required to repeat words either in the same direction (forward repetition) or in reverse direction (backward repetition). For backward repetition, instructions indicated that responses had to include all phonemes, from last to first. The forward repetition task involved 80 items (40 words, 40 pseudowords) presented in increasing length. The mean duration of stimuli for this task was 0.582 s (*SD* = 0.168). For its part, the backward repetition task comprised 120 stimuli (40 words presented in typical order [e.g., *carta*, meaning ‘letter’], 40 words presented in reverse order (e.g., *efej* /efej̃/ [for *jefe* /j̃efe/]*,* meaning ‘boss’), and 40 pseudowords (e.g., *baisa* /baɪsa/), presented also in increasing length. The mean duration of stimuli for this task was 0.631 s (*SD* = 0.194). The sets used for both tasks contained 20 high-frequency words; 20 low-frequency words, 20 pseudowords composed of high-frequency syllables; and 20 pseudowords made up of low-frequency syllables. Additionally, the backward repetition task included another 40 words presented in reserve order, with 20 high-frequency and 20 low-frequency items. The use of increasingly long stimuli allowed examining the subjects’ reversing span.

The words used in the forward and backward tasks did not significantly differ in terms of mean frequency [log frequency; forward task: M = 1.34, *SD* = 0.638; backward task: M = 1.36, *SD* = 0.633; *t*(118) =  − 0.176, *p* = 0.860], familiarity [forward task: M = 5.52, *SD* = 0.800; backward task: M = 5.40, *SD* = 0.877; *t*(118) = 0.727, *p* = 0.469], imageability [forward task: M = 4.90, *SD* = 1.16; backward task: M = 4.83, *SD* = 1.29; *t*(118) = 0.291, *p* = 0.772] or concreteness [forward task: M = 4.89, *SD* = 1.08; backward task: M = 4.88, *SD* = 1.10; *t*(118) = 0.042, *p* = 0.966]. Also, the frequency of high-frequency (*M* = 1.93, *SD* = 0.284) and low-frequency (*M* = 0.755, *SD* = 0.164) words was significantly different [*t*(94.23) = 27.76, *p* < 0.001]. Crucially, however, both lists were similar in familiarity [*t*(118) = 1.06, *p* = 0.289] and imageability [*t*(118) =  − 1.74, *p* = 0.085]. Note that the use of different matched lists guarantees that performance on forward and backward tasks is completely independent but still directly comparable. Word properties were extracted from EsPal, a validated database containing psycholinguistic norms for thousands of Spanish words^79^. Pseudowords were extracted from a previous report^47^.

Within-subject comparisons between backward repetition of words and pseudowords was based on a subsets of 30 items per list (10 with two syllables, 10 with three syllables, 10 with four/five syllables), matched for numbers of syllables (words: *M* = 3.03, *SD* = 0.890; pseudowords: *M* = 3.00, *SD* = 0.830; *t*(58) = 0.150, *p* = 0.881) and phonemes (words: *M* = 7.40, *SD* = 1.97; pseudowords: *M* = 8.23, *SD* = 2.09; *t*(58) =  − 1.585, *p* = 0.118).

Each trial began with a white fixation cross appearing for 300 ms over a black screen, followed by the auditory stimulus (which was administered during the display of the black screen). The maximum response time given before the start of the following trial was 3 s for forward and 7 s for backward trials. Subjects’ responses were audio-recorded and then examined individually by one of the authors. Only responses that completely matched the expected answer (i.e., those that matched all the phonemes presented, in either forward or backward direction) were categorized as correct and given 1 point. Otherwise, responses were categorized as incorrect and given 0 points.

Forward and backward sentence repetition: The forward (list 1) and backward sentence repetition (list 2) tasks were used to assess phoneme sequencing ability in both normal and reserve order, but at the sentential level. Each of these tasks consisted of 25 sentences of increasing length (with five-trial sets comprising sentences of four, six, eight, ten, and 11–12 words). The mean duration of stimuli for the two lists used in this task was 2.436 s (*SD* = 0.923) for list 1 and 2.416 s (*SD* = 0.844) for list 2. Here, too, the use of increasingly long sentences allowed assessing the subjects’ reversing span. Sentences for both lists had similar grammatical structure (all were declarative, indicative, affirmative, unmarked, and endowed with one coordinated phrase), same number of words (*n* = 197), frequency of contained words (log frequency; list 1: *M* = 2.70, *SD* = 0.1.45; list 2: *M* = 2.66, *SD* = 1.50) [*t*(392) = 0.323, *p* < 0.747] and mean number of phonemes in contained words (list 1: *M* = 4.60, *SD* = 2.96; list 2: *M* = 4.62, *SD* = 2.95) [*t*(392) = 0.034, *p* < 0.973].

Stimuli for the backward and forward language tasks were presented binaurally through Panasonic on-ear stereo headphones through an PC running with Windows 7. All stimuli were created using the text-to-speech (TTS) function included by default in Mac computers (version 10.11) and delivered through Psychtoolbox on Matlab software (v. 2016b) in .wav format, with a sampling rate of 44,100 Hz. The volume of presentation was adjusted for each subject individually. In each task, the proportion of correct responses for each subject was calculated and compared to the mean proportion obtained by the control group.

Lexical decision task: This task was used to assess lexical access for words presented in conventional and reversed order. It comprised 96 stimuli, namely: 24 words in typical order, 24 words in reverse order, and 48 pseudowords. Participants were instructed to press the “yes” key when a real word appeared on the screen (regardless of whether it was spelt forwards or backwards) and to press the “no” key when a pseudoword appeared. Stimuli remained on the screen for 300 ms and subjects had up to 2.5 s to respond. The inter-stimulus interval varied randomly between 1.5 and 2.5 s. Words written forward and backward were similar in mean frequency [*t*(45.54) = 0.863, *p* = 0.393], number of phonemes [*t*(46) =  − 0.451, *p* = 0.654], familiarity [*t*(46) =  − 0.948, *p* = 0.348], and imageability [*t*(46) =  − 192, *p* = 0.849]. Pseudowords were created by changing two phonemes to each real-word stimulus. Performance was established by calculating the percentage of correct responses (accuracy) and the response times (RTs) in correct items. For RT measures, values occurring 2.5 *SD*s above or below the participants’ means were considered outliers and removed from the analysis.

### Neuroimaging data acquisition

MRI acquisition and preprocessing steps are reported following guidelines from the Organization for Human Brain Mapping (OHBM)^48^. Whole-brain T1-weighted anatomical 3D scans, spin echo volumes, were acquired in a 1.5-T Phillips Intera scanner with a standard eight-channel head coil. Scans were acquired parallel to the plane connecting the anterior and posterior commissures. The acquisition parameters used were: repetition time (TR) = 7.489 ms; echo time (TE) = 3.420 ms; flip angle = 8°; 175 slices, slice thickness = 4 mm; interslice gap = 4 mm; matrix dimension = 256 × 256; voxel size = 1 × 1 × 1 mm^3^; sequence duration = 7 min. Additionally, diffusion-weighted imaging (DWI) were acquired with a twice-refocused, single-shot, echo-planar imaging pulse sequence. The tensor was computed using 32 non-collinear diffusion directions (b = 800 s/mm^2^) that were maximally spread by considering the minimal energy arrangement of point charges on a sphere, and one scan without diffusion weighting (b = 0 s/mm^2^, b0). The following parameters were used: TR = 11,837.40 ms; TE: 75; flip angle = 90°; 65 slices; slice thickness = 2 mm; interslice gap = 2 mm; voxel size was 2 × 2 × 2 mm^3^; sequence duration = 19 min. Finally, we acquired functional MRI resting-state recordings with 33 axial slices. Functional GRE-EPI volumes were registered in sequential ascent, parallel to the anterior–posterior commissures, covering the whole-brain, using single-shot mode and rectilinear k-space filling. The following parameters were used: TR = 2.777 ms; TE = 50 ms; flip angle = 90°; 33 slices, matrix dimension = 64 × 64; voxel size in plane = 3.6 × 3.6 mm; slice thickness = 4 mm; interslice gap = 4 mm; sequence duration = 10 min; number of volumes = 209. Participants were asked to keep their eyes closed and to avoid moving or falling asleep during the acquisition of the functional volumes.

The two backward speakers underwent the complete scanning session. Structural (T1-weighted) images were obtained from all 24 controls, while DWI and resting-state functional connectivity (rsFC) recordings could be obtained only for 18 and 15 of them, respectively. Importantly, the control group used for each analysis remained sociodemographically matched with both subjects (for more details, see Supplementary Information, Sect. 1).

### Neuroimaging analyses

#### Whole-brain voxel-based morphometry (VBM)

Whole brain voxel-based morphometry (VBM) was used to quantify differences in grey matter volume between each backward speaker and the control group^49^. Preprocessing analyses were performed using the computational anatomy toolbox (CAT12: https://www.neuro.uni-jena.de/cat/) for the Statistical Parametric Mapping (SPM12: https://www.fil.ion.ucl.ac.uk/spm/) software, running on Matlab (2016b). First, all T1 weighted images were manually reoriented to set the origin to the anterior commissure using the Reorient function from SPM12. Then, the standard preprocessing pipeline of CAT12 was used. Briefly T1-weighted images were segmented, corrected for signal inhomogeneity and normalized using the Diffeomorphic Anatomic Registration Through Exponentiated Lie algebra algorithm (DARTEL). Then, the corresponding normalization parameters were applied to the segmented gray matter images. Subsequently, the resulting gray matter normalized images were modulated by their Jacobian determinants and spatially smoothed (FWHM = 10 mm), which allow direct comparison of regional differences in the volume of gray matter volume^50^. The total intracranial volume (TIV) was calculated as the sum of the gray matter, white matter, and cerebrospinal fluid. Finally, images were visually inspected.

Statistical analyses were performed on Matlab (2016b). First, the TIV of each participant was regressed out from the intensity of each voxel of the smoothed grey matter images. The residual of this analysis was used for the statistical analysis. A Crawford *t*-test^41^ was used to compare the intensity of the calculated residual images of each of the two backward speakers with the control group. The results were visualized and corrected for false discovery rate (FDR) using xjView toolbox (https://www.alivelearn.net/xjview). The FDR procedure was applied at the voxel level (*p* < 0.005 corrected), as explained in previous works^51^.

#### Tractography: Automatic fiber quantification (AFQ)

##### Diffusion tensor imaging (DTI) preprocessing

DTI preprocessing started by correcting for head motion and eddy current distortions through the FMRIB Diffusion Toolbox (FDT), followed by brain extraction via the Brain Extraction Tool (BET^52^)—both toolboxes are part of the FMRIB Software Library (FSL 5.0.1; www.fmrib.ox.ac.uk/fsl/). Reconstruction of the diffusion tensor was carried out using least-square estimation algorithm included in the Diffusion Toolkit [TrackVis software, Ruopeng Wang, Van J. Wedeen (trackvis.org/dtk), Martinos Center for Biomedical Imaging, Massachusetts General Hospital, Boston, MA]. Whole-brain tractography used an angular threshold of 35 degrees and an FA threshold of 0.15. Finally, FA maps were generated using Diffusion Toolkit for each subject.

##### Automatic dissection of white matter pathways

Automatic virtual dissections were performed using Automated Fiber Quantification software (AFQ, https://github.com/jyeatman/AFQ)^53^. Previous studies have shown that automatic dissection using AFQ shows high agreement with manual dissection of the white matter tracts^54,55^, while affording measures in 100 points along the tracts. Different white matter tracts were selected as pathways of interest due to their implication in phonological processing. Specifically, the three segments of the AF (long, anterior, and posterior) were dissected as dorsal language pathways^16^; while the IFOF, the UF, and the ILF were dissected as ventral language pathways^21^. All tracts were dissected in native space and in both cerebral hemispheres.

The FA maps of BS1, BS2, and the control group were imported into the AFQ software package, running in Matlab (2016b). AFQ processing was implemented following a standard pipeline mainly consisting in three steps^53^. First, whole-brain tractography was created via a deterministic algorithm with a fourth Runge–Kutta path integration method and 1-mm fixed step size^56^. Second, tracts were segmented targeting two regions of interest (ROIs) defined in MNI standard space. Only fibers passing through the two ROIs were assigned to a specific tract. Finally, tract refinement was performed in two steps: initially, the dissected tracts were compared, for each subject, with a probabilistic atlas of white matter tracts^57^, and aberrant streamlines were discarded; then, a filter was applied establishing that streamlines that were spatially deviated 4 *SD*s from the core tract were removed. Thereupon, MD and FA were calculated at 100 equidistant nodes along each of the six tracts of interest in each hemisphere. FA and MD are non-specific global indexes of diffusion. FA reflects the directionality coherence of water molecule diffusion, and it is considered an index of microstructural white matter integrity sensitive to factors such as axonal integrity and density, extent of myelination, fiber diameter, and fiber packing^58,59^. MD reflects the magnitude of diffusion; thus, higher MD values mean higher diffusivity. Generally, lower fiber integrity is characterized by decreased FA and increased MD, although this is likely an oversimplification^60^. Note that MD is a summary measure capturing the average of water diffusion along the three axes of the tensor model in each voxel. Thus, whenever significant MD differences emerged between backward speakers and controls, we disentangled the underlying source of variability by examining axial diffusivity (AD, a measure of water diffusion along the longitudinal axis) and radial diffusivity (RD, a measure of water diffusion along the two perpendicular axes) in the significant nodes. This allowed us to study the statistically significant differences between each backward speaker and the controls in any part of the white matter microstructure, making point-by-point comparisons along the tract. Crawford’s two-tailed *t*-tests^41^ were used to evaluate point-wise comparisons between each backward speaker and the control group. In order to avoid reporting false positives due to the high number of comparisons (100 nodes), the statistical threshold was set to *p* < 0.05, corrected for multiple comparisons using the false discovery rate (FDR) method^61,62^.

#### Seed-to-voxel resting-state functional connectivity (rsFC) analysis

Resting-state fMRI data and the T1-weighted images of the backward speakers and the control group (*n* = 15) were preprocessed on SPM12 following a standard preprocessing pipeline. The preprocessing steps included: AC-PC orientation for the functional and the T1-weighted images, realignment of the functional volumes to the first volume, coregistration between the functional and the structural T1-weighted image, segmentation of the T1-weighted image into different tissues, normalization of the functional and structural images to the MNI space using the parameters derived from the segmentation of the T1-weighted image, and smoothing of the functional volumes with an 8-mm full-width half-maximum kernel.

RsFC analyses were performed with the CONN functional connectivity toolbox v.18a (www.nitrc.org/projects/conn)^63^, through a gold-standard seed-to-voxel approach^64^. Normalized and smoothed functional data were band-pass filtered (0.008–0.09 Hz) to remove low-frequency drift and high-frequency noise effects. The mean timeseries from each seed ROI was used as a predictor in a multiple regression model at each voxel of the brain. Different confound regressors were also included in the model to remove non-interest signals associated to cerebrospinal fluid, white matter, head movement (six motion correction parameters derived from the realignment preprocessing step), and scrubbing. CONN computed the Fisher-transformed bivariate correlation coefficients between the fMRI signal in each ROI (averaged across all voxels within the ROI) and every voxel of the brain, resulting in a connectivity map that represents all other voxels that are correlated with the seed ROI. The seed ROIs were 6-mm spheres selected from the ROIs available in the CONN toolbox, based on the FSL anatomical atlas. The selected ROIs and the coordinates of the center of the spheres were: left IFG (− 50, 29, 9), right IFG (52, 28, 8), left IC (− 37, 2, 1), right IC (38, 3, − 1), left SMG (− 60, − 39, 31), right SMG (62, − 35, 32), left pSTG (62, − 24, 2) and right pSTG (− 63, − 30, 4). The ROIs selected as seeds for the seed-to-voxel analysis corresponded to brain areas known to be involved in phoneme sequencing, phonological encoding, and speech production^11,12,14,65,66^.

Functional connectivity maps for each seed ROI were used for second-level analysis based on one-way ANCOVAs with age as a covariate, as implemented in the CONN toolbox, in order to study differences between each subject and the control group. Considering that analyses were performed for eight seeds (four in each hemisphere), cluster-level FDR correction was adjusted to p < 0.00625 in order to diminish family-wise error rate. This resulted from dividing 0.05 α-value by the eight tested seeds (i.e., Bonferroni correction). Thereby, results are reported using a whole-brain cluster-level FDR correction (*p* < 0.00625) for voxel-wise analyses at *p* < 0.001 (uncorrected)^67^.

## Results

### Behavioral results

#### General cognitive tasks

##### Reasoning test

Results for the Matrix Reasoning subtest showed no significant differences between the backward speakers (standardized score: BS1 = 14, BS2 = 10) and the control group (standardized score: *M* = 13.33; *SD* = 1.81) (BS1: Crawford’s *t*, two-tailed = 0.360; *p* = 0.723; BS2: Crawford’s *t*, two-tailed = -1.79; *p* = 0.091).

##### Memory tasks

Forward and backward digit span: Results from the forward digit span task (BS1 = 16; BS2 = 8; controls = 8.61, range = 6–11) revealed that, relative to controls, performance was higher for BS1 (Crawford’s *t*, two-tailed = 4.67; *p* < 0.001) and similar for BS2 (Crawford’s *t*, two-tailed =  − 0.386; *p* < 0.704). As regards backward digit span (BS1 = 12; BS2 = 8; Controls = 8.28, range = 4–11), no significant differences were observed between backward speakers and controls (BS1: Crawford’s *t*, two-tailed = 1.76; *p* = 0.095; BS2: Crawford’s *t*, two-tailed =  − 0.133; *p* = 0.895).

Operational span task: Three subjects from the control group were excluded from data analysis because they failed to reach the minimum performance level of 85% on the math operations. Both backward speakers successfully reached the criterion. The number of correctly recalled items was 55 for BS1, 11 for BS2, and 50.93 for controls (range 8–65). BS1 did not significantly differ from controls in either correctly recalled items (Crawford’s *t*, two-tailed = 0.379; *p* = 0.710) or math errors (Crawford’s *t*, two-tailed = 0.503; *p* = 0.622). For his part, compared with controls, BS2 recalled fewer items (Crawford’s *t*, two-tailed =  − 3.71; *p* = 0.002) but did not differ in the number of math errors (Crawford’s *t*, two-tailed = -0.951; *p* = 0.357).

Symmetry span task: One subject from the control group was excluded from data analysis because he failed to reach the 85% accuracy criterion on the symmetry judgement trials. Both backward speakers successfully reached the criterion. The number of correctly recalled items was 32 for BS1, 10 for BS2, and 27.59 for controls (range 17–40). Relative to controls, BS1 showed similar performance in both correctly recalled positions (Crawford’s *t*, two-tailed = 0.629; *p* = 0.538) and symmetry errors (Crawford’s *t*, two-tailed = 0.313: *p* = 0.758). Instead, BS2 showed lower performance in correctly recalled positions (Crawford’s *t*, two-tailed = − 2.51; *p* = 0.023), with no differences in the rate of symmetry errors (Crawford’s *t*, two-tailed = 1.01; *p* = 0.323). For more statistical details, see Supplementary Information*,* Sect. 2 (Table S1).

#### Forward and backward language tasks

##### Forward and backward word and pseudoword repetition

**Figure 1:**
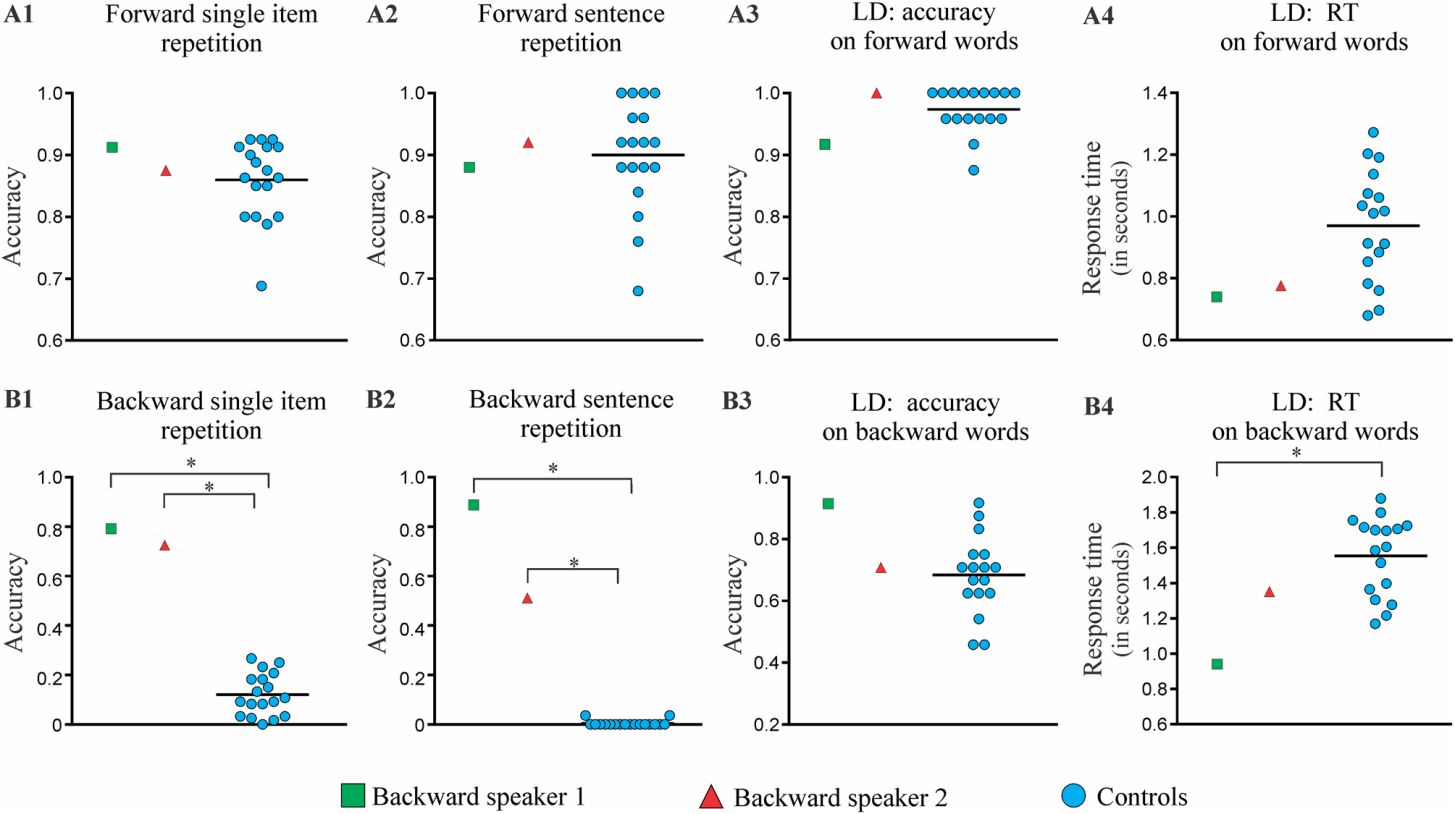
Behavioral results. The figure shows outcomes for the two backward speakers and controls in the repetition and lexical decision (LD) tasks, both in forward (**A1**,**A2**,**A3**,**A4**) and backward (**B1**,**B2**,**B3**,**B4**) direction. The asterisk (*) indicates significant differences at *p* < .05.

The proportion of correctly repeated items in forward repetition was 0.91 for BS1, 0.87 for BS2, and a mean of 0.86 for controls (range 0.68–0.92). Proportions for backward repetition were 0.79 for BS1, 0.73 for BS2, and 0.12 for controls (range 0–0.27). Results from forward repetition revealed no significant differences between the backward speakers and controls for either words (BS1: Crawford’s *t*, two-tailed = 0.000; *p* = 1; BS2: Crawford’s *t*, two-tailed = 0.487; *p* = 0.500) or pseudowords (BS1: Crawford’s *t*, two-tailed = 0.893; *p* = 0.384; BS2: Crawford’s *t*, two-tailed = 0.000; *p* = 1) (Fig. 1, panel A1). Conversely, backward repetition revealed higher accuracy for the two backward speakers than controls in all stimulus types, namely: words presented forwards (BS1: Crawford’s *t*, two-tailed = 5.19; *p* < 0.001; BS2: Crawford’s *t*, two-tailed = 5.94; *p* < 0.001), words presented backwards (BS1: Crawford’s *t*, two-tailed = 5.73 ; *p* < 0.001; BS2: Crawford’s *t*, two-tailed = 4.87; *p* < 0.001), and pseudowords (BS1: Crawford’s *t*, two-tailed = 15.72; *p* < 0.001; BS2: Crawford’s *t*, two-tailed = 11.48; *p* < 0.001) (Fig. 1, panel B1). For more statistical details, see Supplementary Information, Sect. 2 (Tables S2, S3). All results remained significant (i.e., higher accuracy for backward speakers) after controlling for memory abilities (i.e., forward and backward digit span and the operational and symmetry span tasks)–for more statistical details, see Supplementary Information, Sect. 2 (Table S4).This result suggests that backward abilities are not explain by memory skills.

Also, reversal outcomes were higher for words than pseudowords in both subjects. This was true when considering all items in the tasks [BS1: χ^2^(1) = 6.328, *p* = 0.012; BS2: χ^2^(1) = 24.067, *p* < 0.001] and length-matched subsets of 30 items per stimulus type [BS1: χ^2^(1) = 5.88, *p* = 0.015; BS2: χ^2^(1) = 12.604, *p* < 0.001].

##### Forward and backward sentence repetition

The proportion of sentences correctly repeated in forward direction was 0.88 for BS1, 0.92 for BS2, and a mean of 0.90 for controls (range 0.88–1). Proportions in backward repetition were 0.88 for BS1, 0.56 for BS2, and a mean of 0.004 for controls (range 0–0.04). Results from forward sentence repetition task revealed no significant accuracy differences between the two backward speakers and controls (BS1: Crawford’s *t*, two-tailed =  − 0.221; *p* = 0.827; BS2: Crawford’s *t*, two-tailed = 0.221; *p* = 0.827) (Fig. 1, panel A2). Contrariwise, backward sentence repetition proved significantly more accurate for both BS1 (Crawford’s *t*, two-tailed = 65.58; *p* < 0.001) and BS2 (Crawford’s *t*, two-tailed = 41.63; *p* < 0.001) (Fig. 1, panel B2). In fact, the control group showed a floor effect, with only two participants being able to reverse only one short (three-word) sentence. For more statistical details, see Supplementary Information*,* Sect. 2 (Tables S2, S3). Further analyses were performed including each of the four memory tasks as covariates. The accuracy of both subjects was still significantly higher than that of the control group when controlling for memory capacity—for statistical details, see Supplementary Information, Sect. 2 (Table S4). As in the previous task, this result suggests than the ability to reverse sentences is not explained by memory skills.

As regards direct comparisons between both backward speakers, no significant differences emerged between BS1 and BS2 in reversing words presented in forward direction [χ^2^(1) = 1.385, *p* = 0.239], words presented in backward direction [χ^2^(1) = 0.564, *p* = 0.453], or pseudowords [χ^2^(1) = 1.857, *p* = 0.173]. Yet, a significant difference between subjects was observed in backward sentence repetition [χ^2^(1) = 4.861, *p* = 0.027], with BS1 outperforming BS2.

##### Lexical decision task

The proportion of accurate responses was 0.95 for BS1, 0.85 for BS2, and a mean of 0.79 for controls (range 0.66-0.93). Accuracy for words written in forward direction did not differ between controls and either BS1 (Crawford’s *t*, two-tailed =  − 1.35; *p* = 0.195) or BS2 (Crawford’s *t*, two-tailed = 0.810; *p* = 0.429) (Fig. 1, panel A3). Similarly, non-significant differences in RT was observed between controls and BS1 (Crawford’s t, two-tailed =  − 1.23; p = 0.235) or BS2 (Crawford’s t, two-tailed =  − 1.07; p = 0.300) (Fig. 1, panel A4). Accuracy for words written in backward direction was also similar between controls and both BS1 (Crawford’s *t*, two-tailed =  − 1.83; *p* = 0.084) and BS2 (Crawford’s *t*, two-tailed = 0.230; *p* = 0.821) (Fig. 1, panel B3). Contrarily, RT was lower than controls for BS1 (Crawford’s *t*, two-tailed =  − 2.67; *p* = 0.016) but not for BS2 (Crawford’s *t*, two-tailed =  − 0.880; *p* = 0.391) (Fig. 1, panel B4). Finally, accuracy for pseudowords did not differ between controls and either subject (BS1: Crawford’s *t*, two-tailed = 1.74; *p* = 0.099; BS2: Crawford’s *t*, two-tailed = 0.759; *p* = 0.458). Still, this condition yielded lower RT for BS1 (Crawford’s *t*, two-tailed =  − 2.13; *p* = 0.048) but not for BS2 (Crawford’s *t*, two-tailed =  − 1.42; *p* = 0.174). For more statistical details, see Supplementary Information, Sect. 2 (Table S5).

For BS1, no differences were observed in the proportion of accurate responses between words written in forward direction (0.92) and either words in backward direction (0.92) or pseudowords (0.98; χ^2^(1) = 2.632, *p* = 0.104). Yet, RTs were lower in the case of words presented in forward direction (*M* = 0.742, *SD* = 0.133) than in words written in backward direction [*M* = 0.939 ms, *SD* = 0.207; *t*(41) =  − 3.739, *p* = 0.001] and pseudowords [*M* = 0.997 ms, *SD* = 0.195; *t*(66) =  − 5.535, *p* < 0.001]. Contrarily, no significant difference in RTs emerged when comparing words presented backward and pseudowords [*t*(65) =  − 1.098, *p* = 0.276]. For BS2, significantly greater accuracy was observed for words written forward (1) than for both words written in backward direction [0.71; χ^2^(1) = 31.62, *p* < 0.001] and for pseudowords [0.85; χ^2^(1) = 14.13, *p* < 0.001]. Significant lower RTs were observed for forward words (*M* = 0.772 ms, *SD* = 0.110) than for words presented backwards [*M* = 1.35 ms, *SD* = 0.364; *t*(18.1) =  − 6.346, *p* < 0.001] and pseudowords [*M* = 1.24 ms, *SD* = 0.258; *t*(58.8) =  − 10.289, *p* < 0.001]. No significant difference in RTs was observed when comparing words written backward and pseudowords [*t*(22.9) = 1.065, *p* = 0.298].

### Neuroimaging results

**Figure 2:**
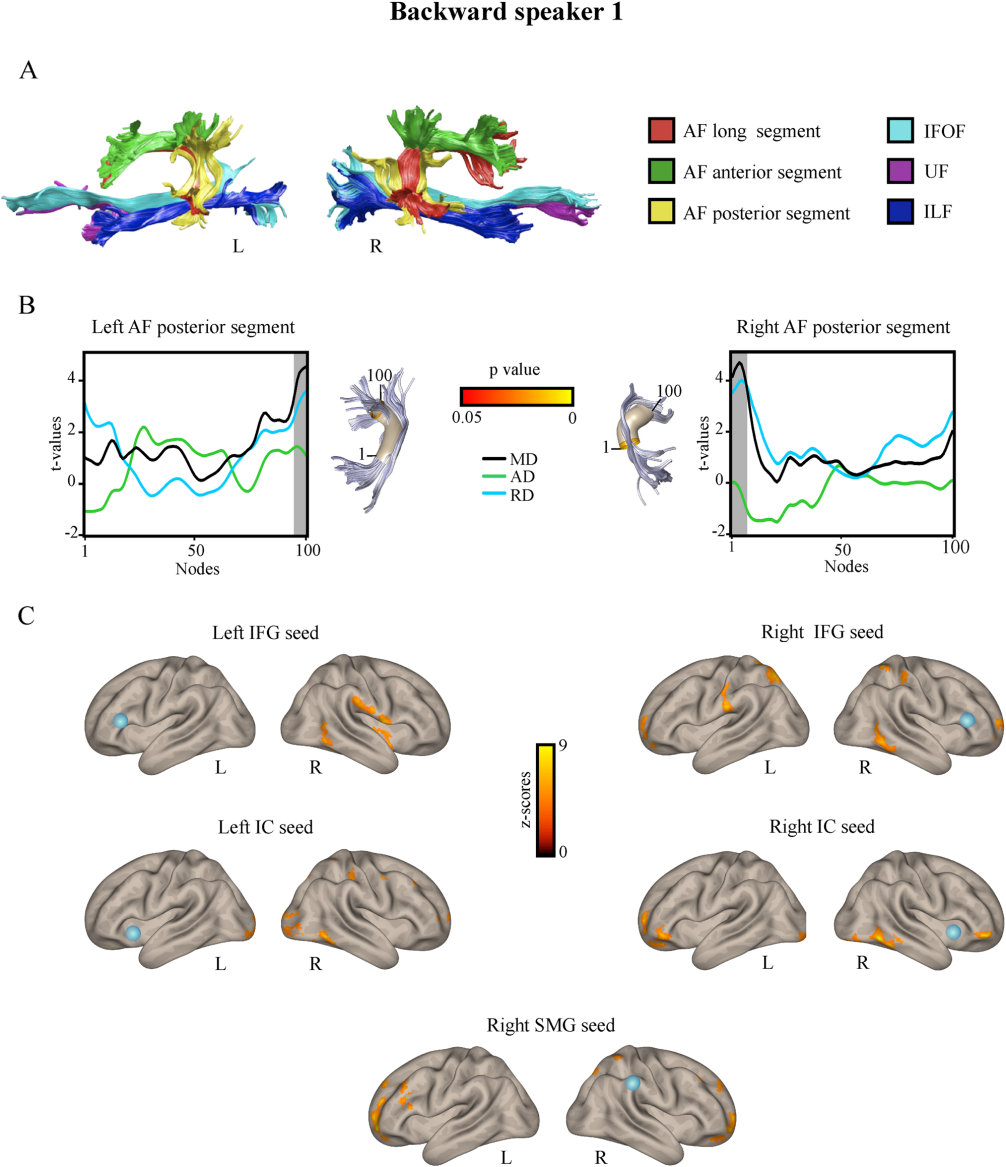
Structural and functional neuroimaging results from Backward speaker 1. (**A**) Deterministic tractography reconstruction of the dorsal and ventral language white matter tracts in Backward speaker 1, in the left and right hemispheres, in native space. (**B**) Tracts profiles showing nodes with significantly different mean diffusivity (MD) in Backward speaker 1 compared to controls (grey shadowed areas). Since MD represents the mean of the three eigenvectors (λ1 + λ2 + λ3/3), in the significant MD clusters, axial diffusivity (AD; λ1) and radial diffusivity (RD, λ2 + λ3/2) indexes were extracted in order to underscore the index driven the change in MD. Note that for the left AF posterior segment both AD and RD are higher, although not significantly, than for controls and, thereby, both indexes seem to contribute to the observed higher MD. For the right AF posterior segment, significantly greater MD is driven by significantly higher RD, as AD shows not significant differences from controls. racts are shown using a three-dimensional rendering derived from Automated Fiber Quantification (AFQ) software, depicting a core fiber represented as a 5-mm-radius tube (color-coded based on t-values at each node along the tract). (**C**) Seed-to-voxel functional connectivity results. Orange-yellow color indicates voxels showing increased functional connectivity with each seed in Backward speaker 1 compared to controls. The seeds are indicated for each analysis with a blue sphere. *L* left, *R* right, *IFG* inferior frontal gyrus, *IC* insular cortex, *SMG* supramarginal gyrus, *AF* arcuate fasciculusm, *IFOF* inferior fronto-occipital fasciculus, *UF* uncinate fasciculus, *ILF* inferior longitudinal fasciculus.

**Figure 3:**
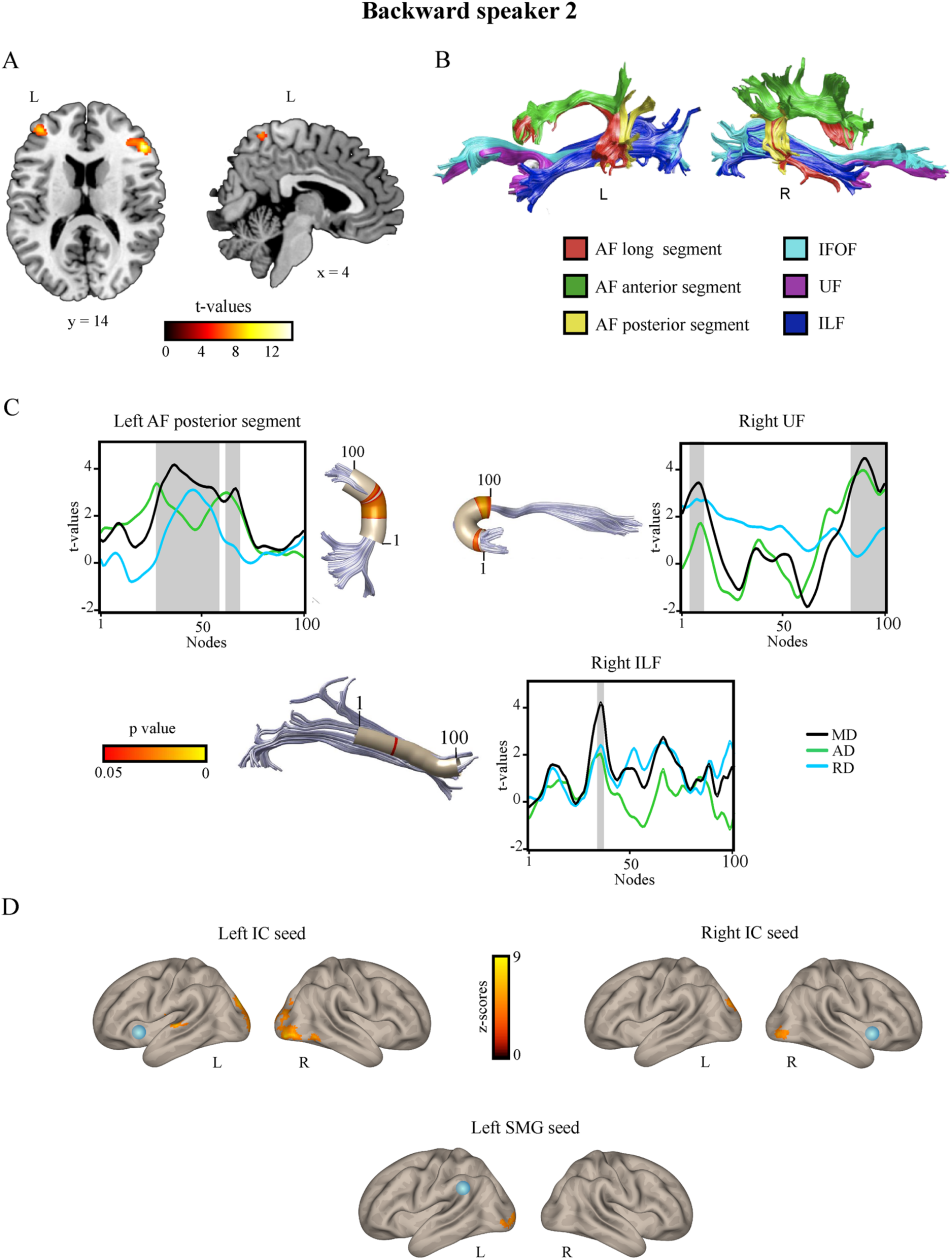
Structural and functional neuroimaging results from Backward speaker 2. (**A**) Voxel-based morphometry (VBM) results. Relative to controls, Backward speaker 2 showed significantly greater grey matter volume (red-yellow colors) in the left inferior and middle frontal gyri and in the right inferior frontal gyrus. Images are shown in standard space over a MNI template available in MRICRON software. (**B**) Deterministic tractography reconstruction of the dorsal and ventral language white matter tracts in Backward speaker 2, in the left and right hemispheres, in native space. (**C**) Tract profiles showing nodes with significant different mean diffusivity (MD) in Backward speaker 2 compared to controls (grey shadowed areas). Since MD represents the mean of the three eigenvectors (λ1 + λ2 + λ3/3), in the significant MD clusters axial diffusivity (AD; λ1), and radial diffusivity (RD, λ2 + λ3/2) indexes were extracted in order to underscore the index driven the change in MD. Note that for the left AF posterior segment, right UF (nodes 5–11) and the ILF both axial diffusivity (AD) and radial diffusivity (RD) are higher, although not significantly, than for controls and, thereby, both indexes seem to contribute to the observed higher MD. For the right UF (nodes 83–100), significantly greater MD is driven by significantly higher AD, while RD shows not significant differences from controls. Nodes are ordered in the dorsal–ventral direction for the AF posterior segment, ventral–dorsal for the UF and posterior–anterior for the ILF. Tracts are shown using a three-dimensional rendering derived from Automated Fiber Quantification (AFQ) software, depicting a core fiber represented as a 5-mm-radius tube (color-coded based on t-values at each node along the tract). (**D**) Seed-to-voxel functional connectivity results. Orange-yellow color indicates voxels showing increased functional connectivity with the seed in Backward speaker 2 compared to controls. The seeds are indicated for each analysis with a blue sphere. *L* left, *R* right, *IC* insular cortex, *SMG* supramarginal gyrus, *AF* arcuate fasciculus, *IFOF* inferior fronto-occipital fasciculus, *UF* uncinate fasciculus, *ILF* inferior longitudinal fasciculus.

Neuroimaging analyses, across volumetric, tractographic, and functional dimensions, revealed potential signatures of the elevated phonemic sequencing skills revealed above for both BS1 (Fig. 2) and BS2 (Fig. 3).

#### Whole-brain voxel-based morphometry (VBM) analysis

A stringent approach to whole-brain VBM results (*p* < 0.005, FDR-corrected) revealed that, compared to controls, BS1 exhibited no significant grey matter volume differences, whereas BS2 showed significantly greater volume in three clusters spanning the bilateral MFG and IFG was well as the right precuneus (Fig. 3A)—for details, see Supplementary Table S7). Nevertheless, based on a less stringent approach (*p* < 0.001, uncorrected, threshold extent = 50 voxels), BS1 showed greater volume than controls in a cluster over the boundary between the left parahippocampal cortex and the FFG region (see Supplementary Fig. S1, Table S6). No significant areas with lower grey matter volume were identified in either subject compared to controls.

#### Tractography results: automatic fiber quantification (AFQ)

Diffusion analysis using AFQ revealed significant differences between both backward speakers and the control group in MD (see Supplementary Tables S8, S9). Differences in FA are not reported as they did not survive the multiple comparison correction—arguably because FA requires larger samples than MD to capture significant effects^68^. Figures 2A and 3B show the tractography reconstruction of the studied tracts for BS1 and BS2, respectively. Relative to controls, BS1 showed higher MD values in nodes of the left (nodes 95–100) and right (nodes 1–7; Fig. 2B) AF posterior segments. For the left AF posterior segment, AD and RD were higher than for controls, although neither of them reached significant differences with controls. For the right AF posterior segment, higher MD was driven by significantly higher RD, while AD showed no significant differences from controls. For his part, compared with controls, BS2 exhibited increased MD in the left AF posterior segment (nodes 29–59 and 63–69), the right UF (nodes 5–11 and 83–100), and the right ILF (nodes 35–37; Fig. 3C). As in BS1, higher MD in nodes of the left AF posterior segment was driven by both higher AD and RD, although, when analyzed separately, none of these indexes reached statistical significance. This was also the case for the right UF (nodes 5–11) and the ILF. Conversely, for the right UF (nodes 83–100), MD differences were driven by significantly higher AD—for details, see Supplementary Information, Sect. 2(Tables S8, S9).

#### Seed-to-voxel resting-state functional connectivity (rsFC) analysis

Results revealed significant differences in functional connectivity between each backward speaker and the control group. For BS1, greater connectivity was found between five seeds and several brain areas (Fig. 2C)—for details, see Supplementary Information, Sect. 2 (Table S10). No seeds were found with significantly decreased functional connectivity. Specifically, compared to controls, BS1 exhibited increased connectivity of (i) the left IFG seed with right dorsal and ventral areas, including part of the SMG, IC, ventral premotor cortex, MTG and ITG; (ii) the right IFG seed with domain-general areas including bilateral parietal, anterior frontal, anterior cingulate and precuneus cortex, in addition to the right MTG and ITG; (iii) the left IC seed with bilateral occipital cortex, bilateral anterior frontal cortex, right cerebellum and right ITG; (iv) the right IC seed with bilateral frontal areas and right posterior ITG; and (v) the right SMG seed with bilateral anterior frontal areas, left MFG and IFG. No significant differences between BS1 and controls were found for the seeds located in the right or left posterior STG, or the left SMG.

BS2 also showed greater connectivity, relative to controls, between specific seeds and, mostly, associative visual posterior regions (Fig. 3D)—for details, see Supplementary Information, Sect. 2 (Table S11). Specifically, stronger connectivity was found between (i) the left IC seed and large clusters involving the left pSTG and bilateral lateral occipital cortex and cuneus; (ii) the right IC seed and the right inferior lateral occipital cortex and bilateral cuneus; and (iii) the left SMG seed and the left occipital pole. Also, decreased connectivity was observed between the left IC seed and clusters involving the left cerebellum. No further significant differences were found.

## Discussion

This study examined neurocognitive signatures of expertise in backward speech as a window into the mechanisms subserving phonological encoding, in general, and phoneme sequencing, in particular. Converging data from behavioral, structural and functional connectivity analyses consistently pointed to components of the dorsal stream (with complementary involvement of ventral, domain-general, and associative-visual mechanisms) as key signatures of elevated phoneme sequencing skills. These results illuminate an understudied dimension of phonological-phonetic encoding while informing models of language-related expertise.

Relative to controls, both subjects had significantly higher accuracy in all backward repetition tasks, with BS1 also exhibiting faster recognition of reversed written words. The fact that neither subject showed advantages in any of the forward repetition tasks suggests that they were specifically gifted for (re)sequencing of phonemes rather than other general phonological operations. Importantly, too, this selective advantage for backward speech remained significant after covarying for outcomes in the verbal and visual span tasks, indicating that the subjects’ behavioral superiority was likely driven by phonological skills proper rather than by general retention abilities, as described for other cases^31^. In line with evidence from other models of expert language processing^69^, this finding suggests that linguistic enhancements due to recurring practice may emerge only for specifically taxed functions, irrespective of other domain-general skills^70^. Furthermore, the observed lexicality effect (better performance in reversing words than pseudowords) suggests that the lexical route may aid reversal abilities, as reported for other phonological processes such as speech repetition^15^. Tentatively, this effect may partly reflect the subjects’ learning of (reversed) phoneme sequences as integrated units, such that their processing as chunks^71^ would lower memory demands and the need for segment-by-segment control^9^. Still, the non-lexical route may also play a role in expert backward processing. Indeed, the lexical decision task indicated that, in both subjects, the time required to access reversed words was similar to that required by pseudowords, both these units proving slower than forward (prototypical) words. Given that pseudowords are usually processed via sub-lexical mechanisms^40^, similar processing time for reversed words might reflect partial reliance on those same mechanisms (although more research would be necessary to test this conjecture).

The subjects’ behavioral advantages were accompanied by specific structural and functional brain patterns. Interestingly, beyond some commonalities, each backward speaker presented distinct neural signatures, suggesting that similar behavioral advantages may result from different plastic adaptations (or from distinctive pre-existing differences) which, in turn, may reflect different underlying strategies^72^. Regarding grey matter volume, comparisons with controls revealed no differences for BS1 under a stringent statistical approach (FDR-corrected, *p* < 0.005). Yet, results from a less exacting but still valid (uncorrected) approach revealed greater grey matter volume in the parahippocampal/FFG, an area involved not only in episodic memory and visuospatial processing^73^, but also in successful verbal memory encoding^74^ and working memory processing^75^. This might constitute a substrate mediating BS1’s greater memory capacity, reflected in his ability to manipulate larger amounts of phonological information—as suggested by his higher performance in reversal of long sentences compared to BS2 and his greater digit span compared to controls. Yet, this result should be taken with extra caution given its more permissive parameters. Instead, more robust volumetric differences were found in BS2, who exhibited greater volume in the bilateral MFG and IFG and the right precuneus under the most stringent (corrected) approach. The bilateral IFG is a critical hub for phonological-phonemic encoding and articulatory planning^76,77^, actually exhibiting increased activation during backward compared to forward speech in expert subjects^31^. Additionally, greater volume in the right precuneus might suggest a complementary involvement of attentional and visual imagery processes in this skill^78^. These particularities reinforce the view that elevated backward speech capacities may be accompanied by diverse neuroanatomical signatures.

As regards white matter differences, backward speakers showed greater MD in several tracts compared to controls. Both subjects exhibited greater MD in parts of the temporo-parietal segment of the AF, bilaterally in BS1 and limited to the left hemisphere in BS2. Although these patterns were driven by higher AD and RD in both subjects, neither index reached significance on its own. Accordingly, higher MD was likely driven by a combination of microstructural changes affecting both AD and RD. On the other hand, higher MD in the right posterior AF of BS1 was driven by significantly greater RD. Tentatively, increased RD may index lower myelinization^79^, or, more plausibly, extra-branching in the nodes involved^80^ (see below for further discussion). Of note, the posterior segment of the AF connects two critical hubs of the phonological network: the posterior STG and the inferior parietal cortex (including the SMG)^16^; indeed, this segment has been linked with different phonological abilities, including repetition^81,82^. Thus, this result hints to a link between the structural configuration of this dorsal tract and phoneme reversal skills. Furthermore, the posterior AF segment reaches posterior inferior and middle temporal areas implicated in orthographic-phonological decoding processes (i.e., visual word-form area)^83^ and in phonological-semantic mappings (i.e., lexical interface)^14^, respectively. Thus, two alternative interpretations may be postulated for these results. First, the distinctive AF pattern observed in both subjects may reflect increased recruitment of the graphemic-phonemic translation circuit^83,84^, sustaining speech reversal through visualization of the to-be repeated stimuli. Second, backward speech may be further supported by direct activation of the reversed phonological sequence through prior activation of the semantic context during speech perception^14,16^. Yet, the latter is unlikely to be the only mechanism supporting this ability, since greater reversal abilities were also observed in meaningless words (i.e., pseudowords). Further research is needed to disentangle among these possibilities.

For his part, BS2 also exhibited significantly greater MD in two differentiated clusters of the right UF (one driven by AD and RD, the other by AD only) and in some nodes of the right ILF (driven by both AD and RD). As part of the ventral pathway, the UF and the ILF participate in semantic processing, lexical retrieval^85,86^, and semantically constrained word learning^54^. Tentatively, this could indicate that inverted words were accessed directly as full lexical units, as suggested above, although further research would be needed to test this conjecture. Alternatively, note that the ventral route can afford compensatory mechanisms when the dorsal stream is overloaded^22^, not developed, or dysfunctional^23–26^. Therefore, the distinctive diffusion pattern found in the UF and ILF may be linked to the systematic recruitment of supporting ventral mechanisms upon excessive demands placed on dorsal routes by continual practice of backward speech. In addition, as the UF is also considered a limbic tract^87^, its particular configuration in BS2 might also be partially associated to the motivational and rewarding value of language reversal, as described for language learning^88^.

Accordingly, the structural differences identified in our subjects may represent putative signatures of their superior abilities for reversing phonological units. Most of these findings fit with previous evidence of structural brain changes associated with expert auditory-motor abilities. Indeed, professional simultaneous interpreters and phoneticians, who exhibit elevated phonemic processing abilities, also show grey matter volumetric (or thickness) increases^89–91^ (but see^92^) and distinctive microstructural white matter properties. In fact, both higher and lower diffusivity in relevant pathways are frequent findings in studies of expert auditory-motor abilities^93–96^. The observed MD adjustments in task-relevant tracts may be attributed to greater efficiency of these networks, potentially resulting from increased axonal caliber, sprouting of collateral branches or greater extracellular space^79,96,97^, affecting to some nodes of the tract. Still, current understanding of diffusion dynamics precludes any fine-grained conclusions in this regard^58,79^.

Additional insights come from our functional connectivity analyses. Compared with controls, both subjects showed greater coupling between phonologically relevant seeds and several areas involved in verbal, visual, and otherwise cognitive processes^98,99^. In particular, the two backward speakers presented enhanced connectivity between the left IC (implicated in articulation^65^ and auditory-motor integration^100^) and right occipito-temporal areas encompassing the FFG region. For BS1, this pattern was also seen for the left and right IFG seeds. Suggestively, given their involvement in orthographic processing, occipito-temporal areas have been proposed to mediate inner visualization strategies during expert backward speech^31^. In fact, meta-analytic evidence attests to the involvement of the bilateral IC in receptive and expressive language processes^101^, whereas the FFG is critically engaged during phonologically demanding tasks^102^ as well as phono-graphemic integration^103^. Moreover, the FFG plays a crucial role in high-level visual processes, such as reading^104,105^, and more critically, due to the increased interaction between FFG and phonological dorsal areas after literacy is attained^106^, it is activated in a top-down manner by phonological stimuli in the absence of visual stimuli^107^. Thus, this coactivation pattern might reflect greater integration between phonological-phonetic encoding and visual-orthographic processes—a possible implicit strategy underlying backward speech skills. However, as proposed below, the specific connectivity patterns observed in each subject suggests that they do not rely on this strategy to an equal extent.

Briefly, BS1 exhibited greater functional coupling between left seeds and several right-sided regions (including perisylvian and occipito-temporal areas), while right seeds showed both greater intra- and inter-hemispheric functional connectivity across perisylvian, parietal, and occipital regions. This pattern of stronger synchrony between language-preferential regions and phonologically sensitive right-sided hubs^66,108^ suggests greater involvement of the right hemisphere in phonological-phonetic encoding. On the other hand, enhanced coupling in the fronto-temporo-parietal network suggests strengthening of circuits involved in auditory-motor integration as well as maintenance and manipulation of verbal information in memory^109,110^—two crucial processes underlying language reversal^31^. Indeed, increased functional coupling between auditory and articulatory areas has been observed in expert simultaneous interpreters^111^, and it is correlated with the ability to learn new verbal material^112^, presumably reflecting elevated phoneme-to-articulation mapping skills. Also, greater connectivity between phonology-related and domain-general areas (i.e., cingulate cortex, prefrontal and parietal areas) may reflect the high cognitive demands of continual backward speech^113^. As regards BS2, all the significant connectivity differences relative to controls pointed to higher coupling between phonologically relevant seeds (IC and SMG) and posterior areas involved in visual and graphemic processing (e.g., occipito-temporal cortex)^114^. Conceivably, this hyperconnectivity pattern between phonological- and vision-related areas might represent a neural fingerprint of implicit visual imagery underlying speech reversal, as previously suggested by regional activation patterns during backward speech^31^. This interpretation, in fact, fits well with the lack of exceptional memory abilities in BS2.

In sum, convergent and divergent results across the two subjects suggest that expertise in phonemic sequencing is mediated by structural and functional adaptations along the dorsal stream, with additional support from ventral, visual-associative, and domain-general areas. This conclusion supports the dual-stream model of language processing^14^ while affording neurocognitive insights on expert language skills. In particular, anatomical and functional adjustment in networks mediating phonological, lexico-semantic, and language control processes have been repeatedly reported in simultaneous interpreters relative to untrained multilinguals^70,115^, and phoneticians relative to subjects without phonetics training^90,96^. The present study extends such findings by showing that language-related neuroplastic adaptations may emerge even for unconventional forms of language expertise, even those that are not publicly used in daily life or honed through professional training.

Finally, as stated earlier, our study indicates that similar forms of language expertise may recruit differential neural mechanisms. Although the majority of the studies on language-related neuroplasticity have favored averaged reports across multiple samples, thus masking potential individual differences within their samples^90,92,93,96^, our results align with few studies using difficult artificial language learning tasks in healthy subjects that report individual variability in the integrity of dorsal and ventral white matter tracts associated to successful learning^22,112^. Crucially, a recent study^116^ has also provided evidence on individual variability in auditory-motor integration abilities, potentially related to the adoption of different cognitive strategies during linguistically demanding tasks. Compatibly, our results suggest that similar forms of language expertise may rely on different neural signatures. Less directly, our results may also have clinical implications, in particular for those disorders characterized by phonemic (or grapheme) phonological encoding or sequencing errors. For instance, conduction aphasics frequently manifest phonemic paraphasias involving phoneme substitution or displacement^117^—a pattern that is also observed in other aphasias types such as the logopenic variant of primary progressive aphasia^118^. In this sense, the identification of critical networks underlying phoneme sequencing skills may promote advances for the diagnosis, prognosis, monitoring, and treatment of such conditions—e.g., by foregrounding key neural targets for non-invasive brain stimulation protocols.

Its contributions notwithstanding, the present study presents some limitations. First, the hallmark of backward speech expertise may conceivably be limited to those neural differences that were shared between both backward speakers (e.g., reshaping of AF), while subject-specific patterns might reflect individual traits (e.g., working memory differences or discrepant processing strategies). Although the present design does now allow for robust testing of these hypotheses, future studies based on larger samples could shed fruitful light on the issue. Second, the present study is based on a small sample size, comprising only two subjects. Although single-case studies have proven crucial for understanding of brain-language relationships, in general^119,120^, and the neural bases of backward speech, in particular^31^, future work should aim to replicate our findings in larger samples. Third, our design did not include online measures of the functional activity during backward speech neural correlates of backward expertise. Even though the results converge with those from task-related fMRI research on spontaneous backward speech^31^, and despite the validity of off-line assessments to detect neural correlates of language expertise^90,93,96^, further investigations involving online and offline neuroscientific measures are needed. Fourth, our cross-sectional design did not allow us to determine whether the neural patterns observed are the result of experience-dependent plastic changes due to training, or if they reflect pre-existing individual differences. Thus, longitudinal studies are required to shed light on this issue. Finally, due to practical limitations, behavioral and neuroimaging analyses were here performed on two separate samples, which precluded the exploration of correlations between performance and neural signatures. Although this methodology has yielded informative results regarding other aspects of language^121^, it would be desirable to circumvent such a limitation in new studies.

In conclusion, our results suggest that expertise in backward speech, as a proxy of elevated phonemic sequencing skills, encompasses varied structural and functional adaptations along the dorsal stream, as well as in components of the ventral stream, visual, and otherwise cognitive processing areas. These findings inform current neurocognitive models of phonological encoding and constrain our understanding of language-related neuroplasticity at large. Further research along these lines may illuminate a hitherto underexplored aspect of verbal processing.

## Supplementary information

Supplementary information

## Data Availability

All demographic, behavioral, and neuroimaging data will be made available upon request.

## References

[1] Dell GS, Burger LK, Svec WR (1997). Language production and serial order: A functional analysis and a model. Psychol. Rev..

[2] Levelt WJM, Wheeldon L (1994). Do speakers have access to a mental syllabary?. Cognition.

[3] Vousden JI, Brown GDA, Harley TA (2000). Serial control of phonology in speech production: A hierarchical model. Cogn. Psychol..

[4] Dell GS (1984). Representation of serial order in speech: Evidence from the repeated phoneme effect in speech errors. J. Exp. Psychol. Learn. Mem. Cogn..

[5] MacKay DG, James LE (2004). Sequencing, speech production, and selective effects of aging on phonological and morphological speech errors. Psychol. Aging.

[6] Laganaro M, Zimmermann C (2010). Origin of phoneme substitution and phoneme movement errors in aphasia. Lang. Cogn. Process..

[7] Martin N, Dell GS (2004). Perseverations and anticipations in aphasia: Primed intrusions from the past and future. Semin. Speech Lang..

[8] Wilshire CE (2002). Where do aphasic phonological errors come from? Evidence from phoneme movement errors in picture naming. Aphasiology.

[9] Hickok G (2014). The architecture of speech production and the role of the phoneme in speech processing. Lang. Cogn. Neurosci..

[10] Price CJ (2010). The anatomy of language: A review of 100 fMRI studies published in 2009. Ann. N. Y. Acad. Sci..

[11] Gelfand JR, Bookheimer SY (2003). Dissociating neural mechanisms of temporal sequencing and processing phonemes. Neuron.

[12] Bohland JW, Guenther FH (2006). An fMRI investigation of syllable sequence production. Neuroimage.

[13] Papoutsi M (2009). From phonemes to articulatory codes: An fMRI study of the role of Broca’s area in speech production. Cereb. Cortex.

[14] Hickok G, Poeppel D (2007). The cortical organization of speech processing. Nat. Rev. Neurosci..

[15] Saur D (2008). Ventral and dorsal pathways for language. Proc. Natl. Acad. Sci. U.S.A..

[16] Catani M, Jones DK, Ffytche DH (2005). Perisylvian language networks of the human brain. Ann. Neurol..

[17] Stark BC (2019). Neural organization of speech production: A lesion-based study of error patterns in connected speech. Cortex.

[18] Pilkington E (2017). Sources of phoneme errors in repetition: Perseverative, neologistic, and lesion patterns in jargon Aphasia. Front. Hum. Neurosci..

[19] Schwartz MF, Faseyitan O, Kim J, Coslett HB (2012). The dorsal stream contribution to phonological retrieval in object naming. Brain.

[20] Han Z (2016). White matter pathway supporting phonological encoding in speech production: A multi-modal imaging study of brain damage patients. Brain Struct. Funct..

[21] Dick AS, Bernal B, Tremblay P (2014). The language connectome. Neuroscience.

[22] López-Barroso D (2011). Language learning under working memory constraints correlates with microstructural differences in the ventral language pathway. Cereb. Cortex.

[23] Torres-Prioris MJ (2019). Repetitive verbal behaviors are not always harmful signs: Compensatory plasticity within the language network in aphasia. Brain Lang..

[24] López-Barroso D, de Diego-Balaguer R (2017). Language learning variability within the dorsal and ventral streams as a cue for compensatory mechanisms in aphasia recovery. Front. Hum. Neurosci..

[25] Brauer J, Anwander A, Friederici AD (2011). Neuroanatomical prerequisites for language functions in the maturing brain. Cereb. Cortex.

[26] Rauschecker AM (2009). Reading impairment in a patient with missing arcuate fasciculus. Neuropsychologia.

[27] Cowan N, Braine MD, Leavitt LA (1985). The phonological and metaphonological representation of speech: Evidence from fluent backward talkers. J. Mem. Lang..

[28] Coltheart M, Glick MJ (1974). Visual imagery: A case study. Q. J. Exp. Psychol..

[29] Jokel R, Conn D (1999). Case study—Mirror reading, writing and backward speech in a woman with a head injury: A case of conversion disorder. Aphasiology.

[30] Cowan N, Leavitt LA (1982). Talking backward: Exceptional speech play in late childhood. J. Child Lang..

[31] Prekovic S (2016). Multidisciplinary investigation links backward-speech trait and working memory through genetic mutation. Sci. Rep..

[32] Seymour PHK, Aro M, Erskine JM (2003). Foundation literacy acquisition in European orthographies. Br. J. Psychol..

[33] Chia LG, Kinsbourne M (1987). Mirror-writing and reversed repetition of digits in a right-handed patient with left basal ganglia haematoma. J. Neurol. Neurosurg. Psychiatry.

[34] Cowan N, Leavitt LA (1987). The developmental course of two children who could talk backward five years ago. J. Child Lang..

[35] Cowan N, Leavitt LA, Massaro DW, Kent RD (1982). A fluent backward Talker. J. Speech Lang. Hear. Res..

[36] Cocchi R, Pola M, Sellerini M, Tosaca P, Zerbi F (1985). Mirror speaking after neurosurgery. A case history. Acta Neurol. Belg..

[37] Mitchell SW (1903). Reversals of habitual motions, backward pronunciation of words, lip whispering of the insane, sudden failures of volition. Repetition impulses. J. Neurol. Ment. Disord..

[38] Chein JM, Ravizza SM, Fiez JA (2003). Using neuroimaging to evaluate models of working memory and their implications for language processing. J. Neurolinguist..

[39] Dehaene S, Le Clec’h G, Poline J-B, Le Bihan D, Cohen L (2002). The visual word form area: A prelexical representation of visual words in the fusiform gyrus. NeuroReport.

[40] Coltheart M, Besner D, Jonasson JT, Davelaar E (1979). Phonological encoding in the lexical decision task. Q. J. Exp. Psychol..

[41] Crawford JR, Garthwaite PH, Howell DC (2009). On comparing a single case with a control sample: An alternative perspective. Neuropsychologia.

[42] Crawford JR, Garthwaite PH, Ryan K (2011). Comparing a single case to a control sample: Testing for neuropsychological deficits and dissociations in the presence of covariates. Cortex.

[43] Wechsler, D. *WAIS-III: Test de Inteligencia Para Adultos de Wechsler*. (Paidós, 2002).

[44] Unsworth N, Heitz RP, Schrock JC, Engle RW (2005). An automated version of the operation span task. Behav. Res. Methods.

[45] Foster JL (2014). Shortened complex span tasks can reliably measure working memory capacity. Mem. Cogn..

[46] Unsworth N, Redick TS, Heitz RP, Broadway JM, Engle RW (2009). Complex working memory span tasks and higher-order cognition: A latent-variable analysis of the relationship between processing and storage. Memory.

[47] Aguado-Alonso, G. Contribuciones al diagnóstico del trastorno específico del lenguaje por medio de la repetición de pseudopalabras. (2011).

[48] Nichols TE (2017). Best practices in data analysis and sharing in neuroimaging using MRI. Nat. Neurosci..

[49] Ashburner J, Friston KJ (2000). Voxel-based morphometry—The methods. Neuroimage.

[50] Mechelli A, Price C, Friston K, Ashburner J (2005). Voxel-based morphometry of the human brain: Methods and applications. Curr. Med. Imaging Rev..

[51] Genovese CR, Lazar NA, Nichols T (2002). Thresholding of statistical maps in functional neuroimaging using the false discovery rate. Neuroimage.

[52] Smith SM (2002). Fast robust automated brain extraction. Hum. Brain Mapp..

[53] Yeatman JD, Dougherty RF, Myall NJ, Wandell BA, Feldman HM (2012). Tract profiles of white matter properties: Automating fiber-tract quantification. PLoS ONE.

[54] Ripollés P (2017). Strength of temporal white matter pathways predicts semantic learning. J. Neurosci..

[55] Vaquero L, Rodríguez-Fornells A, Reiterer SM (2016). The left, the better: White-matter brain integrity predicts foreign language imitation ability. Cereb. Cortex.

[56] Mori S, Crain BJ, Chacko VP, Van Zijl PCM (1999). Three-dimensional tracking of axonal projections in the brain by magnetic resonance imaging. Ann. Neurol..

[57] Hua K (2008). Tract probability maps in stereotaxic spaces: Analyses of white matter anatomy and tract-specific quantification. Neuroimage.

[58] Beaulieu C (2002). The basis of anisotropic water diffusion in the nervous system—A technical review. NMR Biomed..

[59] Pierpaoli C, Basser PJ (1996). Toward a quantitative assessment of diffusion anisotropy. Magn. Reson. Med..

[60] Soares JM, Marques P, Alves V, Sousa N (2013). A hitchhiker’s guide to diffusion tensor imaging. Front. Neurosci..

[61] Benjamini Y, Hochberg Y (1995). Controlling the false discovery rate: A practical and powerful approach to multiple testing. J. R. Stat. Soc. Ser. B.

[62] Multiple Testing Toolbox—File Exchange: MATLAB Central. https://es.mathworks.com/matlabcentral/fileexchange/70604-multiple-testing-toolbox. Accessed 3 May 2020.

[63] Whitfield-Gabrieli S, Nieto-Castanon A (2012). *Conn*: A functional connectivity toolbox for correlated and anticorrelated brain networks. Brain Connect..

[64] Cole DM, Smith SM, Beckmann CF (2010). Advances and pitfalls in the analysis and interpretation of resting-state FMRI data. Front. Syst. Neurosci..

[65] Baldo JV, Wilkins DP, Ogar J, Willock S, Dronkers NF (2011). Role of the precentral gyrus of the insula in complex articulation. Cortex.

[66] Hartwigsen G (2010). Phonological decisions require both the left and right supramarginal gyri. Proc. Natl. Acad. Sci. U.S.A..

[67] Friston KJ, Worsley KJ, Frackowiak RSJ, Mazziotta JC, Evans AC (1994). Assessing the significance of focal activations using their spatial extent. Hum. Brain Mapp..

[68] De Santis S, Drakesmith M, Bells S, Assaf Y, Jones DK (2014). Why diffusion tensor MRI does well only some of the time: Variance and covariance of white matter tissue microstructure attributes in the living human brain. Neuroimage.

[69] Santilli M (2019). Bilingual memory, to the extreme: Lexical processing in simultaneous interpreters. Biling. Lang. Cogn..

[70] García AM, Muñoz E, Kogan B (2019). Taxing the bilingual mind: Effects of simultaneous interpreting experience on verbal and executive mechanisms. Biling. Lang. Cogn..

[71] Segawa J, Masapollo M, Tong M, Smith DJ, Guenther FH (2019). Chunking of phonological units in speech sequencing. Brain Lang..

[72] Noppeney U, Friston KJ, Price CJ (2004). Degenerate neuronal systems sustaining cognitive functions. J. Anat..

[73] Aminoff EM, Kveraga K, Bar M (2013). The role of the parahippocampal cortex in cognition. Trends Cogn. Sci..

[74] Strange BA, Otten LJ, Josephs O, Rugg MD, Dolan RJ (2002). Dissociable human perirhinal, hippocampal, and parahippocampal roles during verbal encoding. J. Neurosci..

[75] Bergmann HC, Rijpkema M, Fernández G, Kessels RPC (2012). Distinct neural correlates of associative working memory and long-term memory encoding in the medial temporal lobe. Neuroimage.

[76] Ferpozzi V (2018). Broca’s area as a pre-articulatory phonetic encoder: Gating the motor program. Front. Hum. Neurosci..

[77] Flinker A (2015). Redefining the role of Broca’s area in speech. Proc. Natl. Acad. Sci. U.S.A..

[78] Cavanna AE, Trimble MR (2006). The precuneus: A review of its functional anatomy and behavioural correlates. Brain.

[79] Beaulieu C (2009). The biological basis of diffusion anisotropy. Diffus. MRI.

[80] Halwani GF, Loui P, Rüber T, Schlaug G (2011). Effects of practice and experience on the arcuate fasciculus: Comparing singers, instrumentalists, and non-musicians. Front. Psychol..

[81] Fridriksson J (2010). Impaired speech repetition and left parietal lobe damage. J. Neurosci..

[82] Forkel SJ (2020). Anatomical evidence of an indirect pathway for word repetition. Neurology.

[83] De Schotten MT, Cohen L, Amemiya E, Braga LW, Dehaene S (2014). Learning to read improves the structure of the arcuate fasciculus. Cereb. Cortex.

[84] Broce IJ (2019). Fiber pathways supporting early literacy development in 5–8-year-old children. Brain Cogn..

[85] Papagno C (2011). Naming and the role of the uncinate fasciculus in language function. Curr. Neurol. Neurosci. Rep..

[86] Basilakos A (2014). Regional white matter damage predicts speech fluency in chronic post-stroke aphasia. Front. Hum. Neurosci..

[87] Von Der Heide RJ, Skipper LM, Klobusicky E, Olson IR (2013). Dissecting the uncinate fasciculus: Disorders, controversies and a hypothesis. Brain.

[88] Ripollés P (2014). The role of reward in word learning and its implications for language acquisition. Curr. Biol..

[89] Becker M, Schubert T, Strobach T, Gallinat J, Kühn S (2016). Simultaneous interpreters vs. professional multilingual controls: Group differences in cognitive control as well as brain structure and function. Neuroimage.

[90] Golestani N, Price CJ, Scott SK (2011). Born with an ear for dialects? Structural plasticity in the expert phonetician brain. J. Neurosci..

[91] Hervais-Adelman A, Moser-Mercer B, Murray MM, Golestani N (2017). Cortical thickness increases after simultaneous interpretation training. Neuropsychologia.

[92] Elmer S, Hänggi J, Jäncke L (2014). Processing demands upon cognitive, linguistic, and articulatory functions promote grey matter plasticity in the adult multilingual brain: Insights from simultaneous interpreters. Cortex.

[93] Elmer S (2019). Tracking the microstructural properties of the main white matter pathways underlying speech processing in simultaneous interpreters. Neuroimage.

[94] Elmer S, Hänggi J, Meyer M, Jäncke L (2011). Differential language expertise related to white matter architecture in regions subserving sensory-motor coupling, articulation, and interhemispheric transfer. Hum. Brain Mapp..

[95] Imfeld A, Oechslin MS, Meyer M, Loenneker T, Jancke L (2009). White matter plasticity in the corticospinal tract of musicians: A diffusion tensor imaging study. Neuroimage.

[96] Vandermosten M, Price CJ, Golestani N (2016). Plasticity of white matter connectivity in phonetics experts. Brain Struct. Funct..

[97] Giacosa C, Karpati FJ, Foster NEV, Penhune VB, Hyde KL (2016). Dance and music training have different effects on white matter diffusivity in sensorimotor pathways. Neuroimage.

[98] Duncan J, Owen AM (2000). Common regions of the human frontal lobe recruited by diverse cognitive demands. Trends Neurosci..

[99] Koechlin E (2011). Frontal pole function: What is specifically human?. Trends Cogn. Sci..

[100] Mutschler I (2009). Functional organization of the human anterior insular cortex. Neurosci. Lett..

[101] Chang LJ, Yarkoni T, Khaw MW, Sanfey AG (2013). Decoding the role of the insula in human cognition: Functional parcellation and large-scale reverse inference. Cereb. Cortex.

[102] Dietz NAE, Jones KM, Gareau L, Zeffiro TA, Eden GF (2005). Phonological decoding involves left posterior fusiform gyrus. Hum. Brain Mapp..

[103] Tan LH, Laird AR, Li K, Fox PT (2005). Neuroanatomical correlates of phonological processing of Chinese characters and alphabetic words: A meta-analysis. Hum. Brain Mapp..

[104] Weiner KS, Zilles K (2016). The anatomical and functional specialization of the fusiform gyrus. Neuropsychologia.

[105] McCandliss BD, Cohen L, Dehaene S (2003). The visual word form area: Expertise for reading in the fusiform gyrus. Trends Cogn. Sci..

[106] López-Barroso D (2020). Impact of literacy on the functional connectivity of vision and language related networks. Neuroimage.

[107] Dehaene S (2010). How learning to read changes the cortical networks for vision and language. Science.

[108] Vigneau M (2011). What is right-hemisphere contribution to phonological, lexico-semantic, and sentence processing?: Insights from a meta-analysis. Neuroimage.

[109] Froudist-Walsh S (2015). Very early brain damage leads to remodeling of the working memory system in adulthood: A combined fMRI/tractography study. J. Neurosci..

[110] Koenigs M, Barbey AK, Postle BR, Grafman J (2009). Superior parietal cortex is critical for the manipulation of information in working memory. J. Neurosci..

[111] Elmer S, Kühnis J (2016). Functional connectivity in the left dorsal stream facilitates simultaneous language translation: An EEG study. Front. Hum. Neurosci..

[112] Lopez-Barroso D (2013). Word learning is mediated by the left arcuate fasciculus. Proc. Natl. Acad. Sci..

[113] Fedorenko E, Duncan J, Kanwisher N (2013). Broad domain generality in focal regions of frontal and parietal cortex. Proc. Natl. Acad. Sci. U.S.A..

[114] Dehaene S, Cohen L (2011). The unique role of the visual word form area in reading. Trends Cogn. Sci..

[115] Hervais-Adelman A, Babcock L (2019). The neurobiology of simultaneous interpreting: Where extreme language control and cognitive control intersect. Biling. Lang. Cogn..

[116] Assaneo MF (2019). Spontaneous synchronization to speech reveals neural mechanisms facilitating language learning. Nat. Neurosci..

[117] Goodglass H, Kohn SE (1992). Diagnosis of conduction aphasia. Conduction Aphasia.

[118] Gorno-Tempini ML (2008). The logopenic/phonological variant of primary progressive aphasia. Neurology.

[119] de Schotten MT (2015). From phineas gage and monsieur leborgne to H.M.: Revisiting disconnection syndromes. Cereb. Cortex.

[120] Dronkers NF, Plaisant O, Iba-Zizen MT, Cabanis EA (2007). Paul Broca’s historic cases: High resolution MR imaging of the brains of Leborgne and Lelong. Brain.

[121] Steeb B (2018). Progressive compromise of nouns and action verbs in posterior cortical atrophy. Front. Psychol..

