# Supplementary material for: Neurocognitive signatures of phonemic sequencing in expert backward speakers
Source: Sci Rep. 2020 Jun 30;10:10621. doi: 10.1038/s41598-020-67551-z (PMC7326922; doi:10.1038/s41598-020-67551-z)
Supplement: Supplementary file 1 — Supplementary information [file 41598_2020_67551_MOESM1_ESM.pdf]

Neurocognitive signatures of phonemic sequencing in expert backward speakers

María José Torres-Prioris[1,2], Diana López-Barroso[1,2], Estela Càmara[3], Sol Fittipaldi[4,5], Lucas Sedeño[5], Agustín Ibáñez[4,5,6,7,8], Marcelo L. Berthier[1], Adolfo M. García[4,5,8,9,10,*]

[1] Cognitive Neurology and Aphasia Unit, Centro de Investigaciones Médico-Sanitarias, Instituto de Investigación Biomédica de Málaga (IBIMA), University of Malaga, Malaga, Spain

[2] Area of Psychobiology, Faculty of Psychology and Speech Therapy, University of Malaga, Malaga, Spain

[3] Cognition and Brain Plasticity Unit, Bellvitge Biomedical Research Institute (IDIBELL), L'Hospitalet de Llobregat, Barcelona, Spain

[4] Universidad de San Andrés, Buenos Aires, Argentina

[5] National Scientific and Technical Research Council (CONICET), Buenos Aires, Argentina

[6] Universidad Autónoma del Caribe, Barranquilla, Colombia

[7] Center for Social and Cognitive Neuroscience (CSCN), School of Psychology, Universidad Adolfo Ibáñez, Santiago, Chile

[8] Global Brain Health Institute, University of California, San Francisco, United States

[9] Faculty of Education, National University of Cuyo (UNCuyo), Mendoza, Argentina

[10] Departamento de Lingüística y Literatura, Facultad de Humanidades, Universidad de Santiago de Chile, Santiago, Chile

*** Corresponding author:** Adolfo M. García, Ph. D., Universidad de San Andrés and CONICET; Vito Dumas 284, B1644BID Victoria, Buenos Aires, Argentina; Phone: (54-11) 4725-7000. E-mail: adolfomartingarcia@gmail.com

Supplementary information

1. Supplementary methods

1.1. Behavioral assessment

Operational span task. In this task letters are used as to-be-remembered stimuli and math operations as distractors. In each trial, participants viewed a letter and then performed a calculation. This sequence was repeated in sets of three to seven trials, varying randomly across participants, with a total of 75 trials. After each set, subjects were presented with an extended list of letters and had to click on the correct ones in their order of appearance. After the recall screen, a feedback screen showed the number of correctly remembered letters and the percentage of correct calculations. Prior to the task, all subjects underwent a three-stage training phase (for details, see[1]. Also, during the math operation training, we calculated the mean time needed to solve the operations. If during testing subjects took more than the mean time plus 2.5 *SD*s, that trial was computed as a speed error. Scores were calculated by summing the number of letters recorded correctly and in the right order (number of correct responses) and summing up the number of mistakes in math operations (speed and accuracy), named math errors. Only subjects with a performance of 85% or higher in the math operations entered the analysis[2].

Symmetry span task: This test resembles the operational span task, with some differences. First, instead of letters, participants are presented with a four-by-four matrix in which different to-be remembered locations are signaled. The number of locations varied from two to five in each set, with a total of 42 trials. Second, instead of performing math operations, subjects had to judge whether a figure was symmetrical in its vertical axis or not. To this end, participants were shown sequences of spatial locations in the matrix, followed by a figure to judge its symmetry; this was followed by the testing phase. As in the operational span task, feedback was offered concerning the number of correct locations remembered and the percentage of correct answers in the symmetry judgement phase. Before testing, all participants completed three practice blocks allowing for familiarization with all phases of the task. Two scores were calculated, namely: the number of correct positions and of symmetry errors. The former was obtained by summing up the number of locations remembered correctly and in the right presentation order; the latter was established by adding the number of accuracy and speed errors committed in the symmetry judgment trials. The criterion to determine speed errors was the same as that used for the operation span task but based on the time employed in the symmetry judgment trials.

1.2. Stimuli

1.2.1. Forward word and pseudoword repetition task

Words forward (*n* = 40)			
High-frequency words		Low-frequency words	
1-2 syllables	3-6 syllables	1-2 syllables	3-6 syllables
Noche	conferencia	peine	mochila
Sala	instituto	pipa	sátira
Llama	ejecución	soñar	manipular
Juez	médico	cubo	deducir
monte	guitarra	clavo	concordia
avión	capacidad	sobra	ardilla
junta	inversión	truco	coyote
pleno	información	pala	abeja
presión	octubre	pliego	conejo
patria	constitución	dogma	demora

Pseudowords (*n* = 40)							
High-frequency syllables				Low-frequency syllables			
2 syll.	3 syll.	4 syll.	5 syll.	2 syll.	3 syll.	4 syll.	5 syll.
ena	conamo	entosame	terablenicia	olu	burrefo	ustiñole	neciglotadia
cote	paesma	deteraco	cosimenlada	chegue	geoncu	denomugue	cosumanfora
esmo	asope	pacósena	indetomapo	osfu	irrolo	marópeno	anquibesido
saén	taensi	menciabiso	analícato	riol	muñeas	tundialaso	Onotánego
decón	brénodi	autidenes	masperamones	bupil	plúzogue	augicumal	cusmipalates

1.2.2. Backward word and pseudoword repetition task

Words forward (*n* = 40)			
High-frequency words		Low-frequency words	
2 syllables	3-5 syllables	2 syllables	3-5 syllables
amor	Solidaridad	blusa	apoyarse
fecha	Argentina	pera	pereza
cuba	Hospital	fiera	carruaje
hotel	Hermano	ansia	cortina
pagar	Protección	sentar	amargura
acto	Existencia	trueno	escoba
carta	Desarrollar	maldad	tortilla
curso	Comisario	ciervo	bailarín
nación	Ministerio	limbo	martillo
vigor	Respiración	pulso	modernidad
Words backward (*n* = 40)			
achul (lucha)	nóisivelet (televisión)	kilo (olik)	zedarnoh (honradez)
efej (jefe)	nóicisopxe (exposición)	ebleum (mueble)	sateirotsih (historietas)
reac (caer)	ainoloc (colonia)	opas (sapo)	olugnátcer (rectángulo)
arim (mira)	olucríc (círculo)	ralloc (collar)	adalasne (ensalada)
rirba (abrir)	nóisaerc (creación)	nótob (botón)	agutrot (tortuga)
nóinu (unión)	akigól (lógica)	atnam (manta)	anaznam (manzana)
evalc (clave)	otisóporp (propósito)	azat (taza)	ogolónom (monólogo)
eplog (golpe)	ovituceje (ejecutivo)	zeun (nuez)	orenillag (gallinero)
agrac (carga)	orejnartxe (extranjero)	oidet (tedio)	dadineres (serenidad)
apluc (culpa)	ojabart (trabajo)	resoc (coser)	aimedipe (epidemia)

pseudowords (*n* = 40)							
high-frequency syllables				low-frequency syllables			
2 syll.	3 syll.	4 syll.	5 syll.	2 syll.	3 syll.	4 syll.	5 syll.
mengo	diconcias	bacompiter	padestamendos	dentu	rijundios	fubelporón	dolirtagentas
bledos	meterción	atelación	senociaresca	clegas	gosandión	acichesión	fomosiarinda
pronda	camendo	grancodesta	aidespeconte	blismu	putelcho	prantecolde	aicaldisempa
baisa	prestona	perlitebles	elestramienda	zaiña	plasquice	yerguimeblos	esontrafielda
Miendo	puesticón	maprósedas	prodalesciones	quieslo	luesbicán	mafrínegas	cletufansiolas

1.2.3. Forward and backward sentence repetition

List 1

1. Iba andando y cayó
2. Estará lunes y martes
3. Abren escuelas y hospitales
4. Sabe leer y escribir
5. Colgar carteles y avisos
6. Hoy he comido pollo y papas
7. Lo operaron y se recuperó rápido
8. Las opciones parecen buenas y acertadas
9. Multan por alta velocidad y drogas
10. La lectura es gratificante y divertida
11. Aprobé todos los exámenes y pasé de año
12. En esta zona llueve en invierno y verano
13. Ha llegado esta mañana y regresa el sábado
14. Me podría haber llamado y no lo hizo
15. Presenta una obra apta para chicos y grandes
16. Pronto cumple cincuenta años y lo festejará a lo grande
17. La selección de películas incluye algunas clásicas y otras modernas
18. Las computadoras se usan para resolver problemas y para entretenerse.
19. Encima de mi escritorio está la carta y el sello
20. Pedile a la vecina un poco de leche y chocolate
21. Hubo varios heridos en un accidente y llamaron a la ambulancia
22. Son características típicas de los monos y de los humanos inteligentes
23. El ejercicio físico mejora la salud y el estado de ánimo general
24. El auto que me he comprado lleva termómetro y equipo de música
25. El cerebro controla funciones vitales y permite responder a estímulos externos

List 2

1. Inventar letras y cantar
2. Es atento y gracioso
3. Luna llena y playa
4. Apagamos y nos vamos
5. Cosas locas y aburridas
6. El escarabajo azul y la tortuga
7. Suena el teléfono y enseguida aparece
8. Esperando su paquete y el cambio
9. Su trabajo exige esfuerzo y paciencia
10. Hay empanadas al horno y canelones
11. Canciones que te traen recuerdos y te alegran
12. Me pidió permiso para salir y se fue
13. Mafalda es una niña muy inteligente y ocurrente
14. El universo está formado por planetas y estrellas
15. El abogado sabe mucho de leyes y sentencias
16. El libro es ameno y cambiará tu forma de pensar
17. Es una idea genial y seguro que lo podés conseguir
18. Cuando era joven jugaba al trompo y a las bolillas
19. Pronto cumplirá su promesa y estaremos todos listos para festejar
20. Los avances tecnológicos han impulsado la industria y la comunicación
21. Los animales característicos de la sabana son la jirafa y el león
22. Las aves revolotean entre los presentes y se posan en las estatuas
23. El taxi paró tres cuadras después y todos bajaron de prisa
24. Todas las semanas salgo a correr y a andar en bicicleta
25. El departamento tiene cuatro habitaciones arriba y un patio amplio abajo

1.2.4. Lexical decision

Words (n = 48)				Pseudowords (n = 48)			
roca	teléfono	Gorriones	jarra	racu	tolófena	garriones	jurro
subir	teniente	Lechuga	morder	sibor	taneinto	lichuzo	murdor
guión	imaginación	Cerrojo	moño	goeún	imigenución	cerrrujo	muña
Tela	solitario	zanahoria	loba	tile	sulitereo	zapatoria	lubo
pierna	fotografía	gaseosa	fresa	poerno	futogrefía	gesiosa	fruso
vaca	espejo	Paella	jarrón	vocu	Espaje	puello	jorrán
guardia	ceremonia	Chorizo	clavel	gaurdio	cirumonea	churoza	cleval
regla	sabiduría	Butaca	barman	rugle	sabudaría	botuca	bermon
sal	esfera	bromear	cartón	sil	esforo	brumeor	cartán
juicio	Anillo	amabilidad	aula	joiceo	anulla	amobiludad	aola
cantar	médica	Anotar	hacha	contur	Mídeco	anuter	hucho
viaje	esquina	Arruga	uña	veaja	esqueno	arragu	eña

2.3. Neuroimaging data acquisition

Tractographic analyses were made with a control sample of 18 participants. This subsample was matched with both backward speakers in terms of age (Backward speaker 1: Crawford's t, two-tailed = .603; p = .554; Backward speaker 2: Crawford's t, two-tailed = 1.025; p = .319) and years of education (Backward speaker 1: Crawford's t, two-tailed = .926; p = .367; Backward speaker 2: Crawford's t, two-tailed = 0.00; p = 1).

Resting-state functional connectivity analyses included a control sample of 15 participants, who were also matched with both backward speakers in terms of age (Backward speaker 1: Crawford's t, two-tailed = .476; p = .641; Backward speaker 2: Crawford's t, two-tailed = .875; p = .396) and years of education (Backward speaker 1: Crawford's t, two-tailed = .602; p = .556; Backward speaker 2: Crawford's t, two-tailed = -.303; p = .767).

2. Supplementary results

Table S1. Performance of the two backward speakers and the control group in cognitive tests.

Task (*n*)	Controls (mean and *SD*)	BS1	BS2	Statistical comparison: BS1 vs. controls				BS2 vs. controls			
				t	*p*	zCC	CI	*t*	*P*	zCC	CI
Matrix reasoning (18)	13.33 (1.81)	14	10	.360	.723	.370	-.113 to .843	-1.79	.091	-1.84	-2.59 to -1.06
Forward digit span (18)	8.61 (1.54)	16*	8	4.67	< .001	4.79	3.13 to 6.45	-.386	.704	-.396	-.871 to .090
Backward digit span (18)	8.28 (2.05)	12	8	1.76	.095	1.81	1.04 to 2.56	-.133	.895	-.137	-.599 to .330
Operational span (15)											
Total number correct	50.93 (10.41)	55	11*	.379	.710	.391	-.141 to .911	-3,71	.002	-3.83	-5.32 to -2.33
Math error s	6.27 (3.33)	8	3	. 503	.622	.520	-.029 to 1.05	-.951	.357	-.982	-1.59 to -.349
Symmetry span (17)											
Total number correct	27.59 (6.81)	32	10*	.629	.538	. 648	.114 to 1.16	-2.51	.023	-2.58	-3.58 to -1.56
Symmetry errors	2.47 (1.46)	2	4	.313	.758	.322	-.805 to .171	1.01	.323	1.04	.441 to 1.63

n: number of subjects included after removal of outliers and/or data from subjects with a performance below 85% (for the operational and symmetry span tasks). BS1: Backward speaker 1; BS2: Backward speaker 2. Statistical comparisons are based on two-tailed Crawford's *t*-tests[3,4]. Asterisks (*) indicate significant differences at $p < .05$.

Table S2. Accuracy of the two backward speakers and the control group in the performance of four repetition tasks.

Repetition direction	Stimulus type (dependent measure) [*n* = 18]	Controls (mean and *SD*)	BS1	BS2	Statistical comparison							
					BS1 vs. controls				BS2 vs. controls			
					t	*P*	zCC	CI	*t*	*p*	zCC	CI
Forward and backward word and pseudoword repetition												
Forward	Words	.95 (.04)	.95	.97	.000	1	.000	-.462 to .462	.487	.632	.500	.002 to .985
	Pseudowords	.77 (.109)	.87	.77	.893	.384	.917	.354 to 1.46	.000	1	.000	-.462 to .462
Backward	Words forward	.21 (.133)	.92*	1*	5.19	< .001	5.33	3.49 to 7.16	5.78	<.001	5.94	3.90 to 7.96
	Words backward	.13 (.112)	.79*	.69*	5.73	< .001	5.89	3.87 to 7.90	4.87	<.001	5.00	3.26 to 6.72
	Pseudowords	.030 (.039)	.66*	.49*	15.72	< .001	16.15	10.75 to 21.54	11.48	<.001	11.79	7.83 to 15.74
Forward and backward sentence repetition												
Forward	Sentences	.90 (.088)	.88	.92	-.221	.827	-.227	-.692 to .244	.221	.827	.227	-.244 to .692
Backward	Sentences	.00 (.01)	.88*	.56*	65.58	< .001	67.38	44.94 to 89.80	41.63	< .001	42.76	28.25 to 57.00

acc: accuracy. BS1: Backward speaker 1; BS2: Backward speaker 2. Statistical comparison were performed through two-tailed Crawford's *t*-tests[3,4]. Asterisks (*) indicate significant differences at $p < .05$.

Table S3. Accuracy for the two backward subjects in the backward repetition tasks.

	BS1	BS2
Single items[1]		
1-word forward	.92	1
1-word backward	.77	.67
1-pseudoword	.67	.50
**Mean all items**	**.79**	**.72**
Sentences		
4-6 words	1	.90
10-12 words	.70	.30
**Mean all items**	**.88**	**.56**

[1]This task required backward repetition of words presented in forward position (prototypical form), words presented in backward order, and pseudowords. BS1: Backward speaker 1; BS2: Backward speaker 2.

Table S4. Comparison of the two backward speakers with controls in backward tasks, controlling for memory outcomes.

	Statistical comparison[1]					
	BS1 vs. controls			BS2 vs. controls		
	p	zCCC*	CI	*p*	zCCC*	CI
Backward repetition of words and pseudowords	< .001	7.946	3.250 to 10.533	.001	7.153	2.993 to 9.410
Backward repetition of sentences	< .001	67.876	36.674 to 82.031	< .001	43.070	23.177 to 52.111

[1] Bayesian comparison developed to compare a single case's score to that of a control group while controlling for covariates[5]. The BTD_Cov.ex software was used. BS1: Backward speaker 1; BS2: Backward speaker 2. (*) effect size.

Table S5. Performance of the two backward subjects and the control group in the lexical decision task.

Stimulus type and dependent measure (n = 17)	Controls (mean and *SD*)	BS1	BS2	Statistical comparison							
				BS1 vs. controls				BS2 vs. controls			
				t	*p*	zCC	CI	*T*	*p*	zCC	CI
Global accuracy	.79 (.08)	.95	.85	1.94	.069	2.00	1.15 to 2.82	.729	.476	.750	.200 to 1.28
Global RT	1.44 (.257)	.920	1.12	-1.98	.064	-2.04	-2.88 to -1.18	-1.199	.248	-1.23	-1.86 to -.586
Forward word accuracy	.97 (.036)	.92	1	-1.35	.195	-1.38	-2.05 to -.705	.810	.429	.833	.269 to 1.37
Forward word RT	.969 (.179)	.742	.772	-1.23	.235	-1.26	-1.90 to -.613	-1.07	.300	-1.10	-1.69 to -.483
Backward word accuracy	.68 (.127)	.92	.71	1.83	.084	1.89	1.07 to 2.68	.230	.821	.236	-.250 to .715
Backward word RT	1.55 (.222)	.939*	1.35	-2.67	.016	-2.75	-3.80 to -1.68	-.880	.391	-.905	-1.46 to -.327
Pseudoword accuracy	.75 (.128)	.98	.85	1.74	.099	1.79	1.00 to 2.56	.759	.458	.781	.226 to 1.31
Pseudoword RT	1.73 (.335)	.997*	1.24	-2.13	.048	-2.20	-3.08 to -1.29	-1.42	.174	-1.45	-2.13 to -.754

RT: response time in seconds. BS1: Backward speaker 1; BS2: Backward speaker 2. Statistical comparison were performed via Crawford's two-tailed *t*-tests[3,4]. The asterisks (*) indicate significant differences at $p < .05$.

Table S6. Voxel-based morphometry (VBM) results for Backward speaker 1. Results are reported at p <.001, uncorrected, with a minimum threshold extent of 50 voxels.

Cluster size	Peak MNI coordinates *x*	*y*	*z*	Hemisphere	Anatomical label (*n* of voxels)	Peak *t*
**61**	-34.5	-40.5	-7.5	Left	Parahipopcampal (45)<br>Fusiform (12)	4.93

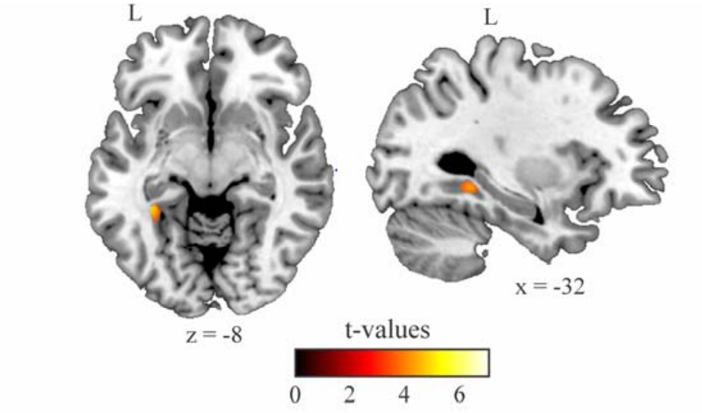

Figure S1. Voxel-based morphometry (VBM) results. Based on an uncorrected threshold of $p < .001$, with a minimum threshold extent of 50 voxels, Backward speaker 1 showed significantly greater grey matter volume (red-yellow colors) than controls in a cluster over the boundary between the left parahippocampal cortex and the fusiform gyrus. Images are shown in standard space over a MNI template available in MRICRON software.

Table S7. Voxel-based morphometry (VBM) results for Backward speaker 2.

Cluster size	Peak MNI coordinates *x*	*y*	*z*	Hemisphere	Anatomical label (*n* of voxels)	Peak *t*
**Significant results at FDR-corrected, *p* ≤ .005**						
**322**	48	33	12	Right	Inferior frontal (290)<br>Middle frontal (29)	15.9
**124**	-37.5	46.5	13.5	Left	Middle frontal (115)<br>Inferior frontal (9)	9.07
**66**	4.5	-54	66	Right	Precuneus (65)	7.22
**Significant results at uncorrected *p* < .001 (minimum extent threshold: 50 voxels)**						
**613**	48	33	12	Right	Inferior frontal (495)<br>Middle frontal (113)	15.9
**372**	-37.5	46.5	13.5	Left	Middle frontal (332)<br>Inferior frontal (40)	9.07
**60**	9	-87	19.5	Right	Cuneus (60)	5.07
**51**	-33	36	43.5	Left	Middle frontal (48)	4.85
**299**	4.5	-54	66	Right	Precuneus (220)	7.22
**72**	39	-45	63	Right	Postcentral (53)<br>Superior parietal (19)	5.18

Table S8. Nodes of mean diffusivity (MD) that significantly differed between Backward speaker 1 (BS1) and the control group. Only results that survived $p < .05$ (FDR-corrected) at the node level are reported. For these nodes, differences in axial (AD) and radial diffusivity (RD) between BS1 and the control group are reported using a $p < .05$ (FDR-corrected) in order to explore the underlying source of variability. Means (*M*) and standard deviations (*SD*) in MD are reported for the control group.

Significant results at FDR-corrected $p \leq 0.05$									
			**Controls**						
**Hemisphere**	**Tract**	**Node**	***M***	***SD***	**BS 1**	***t***	***p (2-tailed)***	***AD*** ***p (2-tailed)***	***RD*** ***p (2-tailed)***
Left	AF posterior segment	95	.76	.036	.89	3.47	.048	.466	.200
		96	.76	.034	.90	3.96	.020	.466	.164
		97	.76	.033	.91	4.26	.013	.466	.134
		98	.76	.033	.91	4.37	.013	.466	.134
		99	.77	.033	.91	4.44	.013	.466	.134
		100	.77	.032	.91	4.50	.013	.466	.134
Right	AF posterior segment	1	.75	.178	.88	4.10	.013	.993	**.037**
		2	.75	.178	.88	4.36	.010	.993	**.032**
		3	.74	.178	.89	4.52	.010	.993	**.032**
		4	.74	.178	.88	4.63	.010	.993	**.032**
		5	.74	.177	.88	4.56	.010	.993	**.032**
		6	.74	.176	.86	4.27	.010	.993	**.032**
		7	.74	.175	.84	3.85	.018	.993	**.032**

AF: arcuate fasciculus. AD and RD nodes that significantly differed between backward speakers and controls (p < .05, FDR-corrected) are highlighted in bold.

Table S9. Nodes of mean diffusivity (MD) that significantly differed between Backward speaker 2 (BS2) and the control group. Only results that survived $p < .05$ (FDR-corrected) at the node level are reported. For these nodes, differences in axial (AD) and radial diffusivity (RD) between BS2 and the control group are reported using a $p < .05$ (FDR-corrected) in order to explore the underlying source of variability. Means (*M*) and standard deviations (*SD*) in MD are reported for the control group.

			**Significant results at FDR-corrected $p \leq 0.05$**						
			**Controls**						
**Hemisphere**	**Tract**	**Node**	***M***	***SD***	**BS 2**	***t***	***p* (2-tailed)**	***AD* p (2-tailed)**	***RD* p (2-tailed)**
Left	AF posterior segment	29	.74	.033	.83	2.71	.033	.102	.990
		30	.74	.033	.84	3.04	.033	.102	.990
		31	.74	.033	.85	3.27	.033	.102	.990
		32	.74	.034	.86	3.42	.034	.102	.917
		33	.74	.035	.87	3.56	.035	.109	.693
		34	.74	.035	.88	3.72	.035	.115	.540
		35	.74	.035	.88	3.89	.035	.119	.429
		36	.74	.035	.88	4.01	.035	.123	.347
		37	.74	.035	.89	4.05	.035	.130	.276
		38	.74	.037	.89	3.99	.037	.135	.230
		39	.74	.038	.89	3.89	.038	.147	.202
		40	.74	.039	.89	3.80	.039	.157	.185
		41	.74	.040	.89	3.73	.040	.171	.172
		42	.74	.041	.89	3.68	.041	.192	.158
		43	.74	.041	.89	3.61	.041	.215	.156
		44	.74	.041	.89	3.53	.041	.231	.156
		45	.74	.042	.89	3.45	.042	.254	.156
		46	.74	.042	.89	3.38	.042	.260	.156
		47	.74	.042	.88	3.32	.042	.266	.156
		48	.74	.042	.88	3.27	.042	.261	.156
		49	.74	.042	.88	3.22	.042	.257	.156
		50	.74	.042	.88	3.19	.042	.231	.158
		51	.74	.041	.88	3.16	.041	.207	.172
		52	.74	.041	.87	3.14	.041	.171	.180
		53	.74	.040	.87	3.13	.040	.152	.185
		54	.74	.040	.87	3.12	.040	.135	.202
		55	.74	.039	.86	3.11	.039	.121	.230
		56	.74	.038	.86	3.06	.038	.112	.276
		57	.74	.038	.85	2.97	.038	.109	.369
		58	.74	.039	.85	2.83	.039	.102	.512
		59	.74	.039	.84	2.69	.039	.102	.693
		63	.74	.036	.83	2.62	.048	.102	.990
		64	.74	.034	.83	2.74	.042	.102	.990
		65	.74	.031	.83	2.89	.033	.102	.990
		66	.74	.029	.83	3.03	.026	.102	.990
		67	.74	.028	.83	3.07	.026	.109	.990
		68	.74	.027	.82	2.93	.030	.119	.990
		69	.74	.028	.82	2.64	.047	.135	.990
Right	Uncinate fasciculus	5	.83	.036	.94	2.96	.037	.751	.229
		6	.83	.036	.94	3.10	.033	.626	.229
		7	.83	.038	.95	3.21	.029	.533	.229
		8	.83	.039	.96	3.31	.029	.459	.229
		9	.82	.040	.96	3.33	.029	.365	.229
		10	.82	.041	.96	3.26	.029	.361	.229
		11	.82	.043	.96	3.04	.035	.417	.229
		83	.74	.038	.85	2.88	.041	**.034**	.551
		84	.73	.039	.86	3.20	.029	**.028**	.464
		85	.73	.040	.87	3.45	.027	**.028**	.425
		86	.73	.041	.89	3.67	.021	**.028**	.328
		87	.73	.041	.90	3.87	.017	**.028**	.272
		88	.73	.041	.91	4.06	.015	**.028**	.245
		89	.73	.041	.91	4.24	.015	**.028**	.229
		90	.73	.040	.91	4.33	.015	**.028**	.229
		91	.73	.040	.91	4.32	.015	**.028**	.229
		92	.74	.039	.90	4.20	.015	**.028**	.244

		93	.74	.037	.89	4.01	.015	**.028**	.272
		94	.74	.036	.88	3.81	.018	**.034**	.328
		95	.74	.034	.87	3.56	.024	**.043**	.435
		96	.74	.033	.85	3.27	.029	.053	.543
		97	.74	.032	.84	3.03	.035	.055	.720
		98	.74	.030	.83	2.97	.037	.055	.811
		99	.74	.027	.83	3.11	.033	.055	.811
		100	.74	.025	.83	3.27	.029	.051	.811
Right	Inferior Longitudinal fasciculus	35	.81	.040	.97	3.81	.047	.899	.274
		36	.82	.038	.98	4.07	.047	.899	.274
		37	.82	.037	.96	3.92	.047	.899	.274

AF: arcuate fasciculus. AD and RD nodes that significantly differed between backward speakers and controls ($p < .05$, FDR-corrected) are highlighted in bold.

Table S10. Seed-to-voxel functional connectivity results based on resting-state data from Backward speaker 1.

Seed location (coordinates)	Clusters MNI coordinates<br>*x* *y* *z*	Cluster size	Hemisphere	Anatomical label	Minimum cluster size *FDR*-corrected [$t(13) = 4.22$]
Left inferior frontal gyrus<br>-50 29 9	54 -2 8	461	Right	(Pre)central operculum<br>Insula<br>Heschl's gyrus	262
	58 -60 -10	369	Right	Lateral occipital<br>Inferior temporal<br>Middle temporal	
	58 -30 26	262	Right	Parietal operculum<br>Supramarginal<br>Postcentral	
Right inferior frontal gyrus<br>52 28 8	-20 -52 62	372	Left	Lateral occipital<br>Superior parietal	166
	50 -54 -2	342	Right	Inferior temporal<br>Middle temporal	
	-60 -24 44	313	Left	Postcentral<br>Supramarginal	
	0 20 28	302	Right/left	Anterior Cingulate	
	8 -54 64	246	Right	Superior parietal	
	-26 50 -12	238	Left	Frontal pole	
	32 60 -4	228	Right	Frontal pole	
	-6 -72 38	173	Right/left	Precuneous	
	38 -36 52	166	Right	Postcentral<br>Supramarginal	
Left insular cortex<br>-37 2 1	58 -62 -10	501	Right	Inferior temporal	118
	18 -98 10	411	Right	Occipital pole	
	-12 -102 -10	344	Left	Occipital pole	
	34 64 06	182	Right	Frontal pole	
	52 2 50	173	Right	Precentral gyrus	
	38 44 38	171	Right	Middle frontal gyrus	
	38 -34 40	153	Right	Postcentral	
	32 -84 -20	131	Right	Occipital<br>Fusiform	
	14 -70 36	121	Right	Precuneous	
	28 -48 68	118	Right	Superior parietal	
Right insular cortex<br>38 3 -1	58 -60 -12	688	Right	Inferior temporal	201
	-38 46 -18	507	Left	Frontal pole	
	32 -88 -20	376	Right	Cerebellum Cru1	
	44 48 -8	302	Right	Frontal pole	
	-24 52 -6	213	Left	Frontal pole	
	-26 -92 -16	201	Left	Occipital pole	
Right supramarginal gyrus<br>62 -35 32	-10 -72 28	883	Right/Left	Cuneus<br>Precuneus	147
	-30 56 -6	549	Left	Frontal pole	
	26 58 -6	471	Right	Frontal pole	
	36 34 46	365	Right	Middle frontal	
	-36 32 40	305	Left	Middle frontal	
	26 -50 68	153	Right	Superior parietal	
	-54 26 16	147	Left	Inferior frontal	

Table S11. Seed-to-voxel functional connectivity results based on resting-state data from Backward speaker 2.

Seed anatomical label (coordinates)	Contrast	Clusters MNI coordinates *x y z*	Cluster size	Hemisphere	Anatomical label	Minimum cluster size *FDR*-corrected [$t(13) = 4.22$]
Left insular cortex -37 2 1	+	38 -92 -12	2567	Right/Left	Occipital pole	188
					Lateral occipital	
					Cuneus	
				Right	Inferior temporal	
				Right	Fusiform	
		-62 -20 4	188	Left	Posterior Superior temporal	
	-	-38 -50 -56	223	Left	Cerebellum	188
Right insular cortex 38 3 -1	+	-2 -72 18	810	Right/Left	Cuneus	165
		38 -84 -6	165	Right	Lateral occipital	
Left supramarginal gyrus -60 -39 31	+	-30 -98 -6	182	Left	Occipital pole	182

Supplementary references

1. Unsworth, N., Redick, T. S., Heitz, R. P., Broadway, J. M. & Engle, R. W. Complex working memory span tasks and higher-order cognition: A latent-variable analysis of the relationship between processing and storage. *Memory* **17**, 635–654 (2009).
2. Unsworth, N., Heitz, R. P., Schrock, J. C. & Engle, R. W. An automated version of the operation span task. *Behav. Res. Methods* **37**, 498–505 (2005).
3. Crawford, J. & Howell, D. C. Comparing an Individual's Test Score Against Norms Derived from Small Samples. *Clin. Neuropsychol.* **12**, 482–486 (1998).
4. Crawford, J. R., Garthwaite, P. H. & Porter, S. Point and interval estimates of effect sizes for the case-controls design in neuropsychology: Rationale, methods, implementations, and proposed reporting standards. *Cogn. Neuropsychol.* **27**, 245–260 (2010).
5. Crawford, J. R., Garthwaite, P. H. & Ryan, K. Comparing a single case to a control sample: Testing for neuropsychological deficits and dissociations in the presence of covariates. *Cortex* **47**, 1166–1178 (2011).
